# A Flame-Retardant Hydrogen-Bonded Organic Framework Separator for Selective Sodium-Ion Transport toward a NaF-Rich Interphase in Sodium Metal Batteries

**DOI:** 10.1007/s40820-026-02160-5

**Published:** 2026-04-09

**Authors:** Muhammad Ali, Moazzam Ali, Hamid Hussain, Samia Aman, Ufra Naseer, Asif Mahmood, Muhammad Tayyab Ahsan, Yinzhu Jiang, Muhammad Yousaf

**Affiliations:** 1 School of Materials Science and Engineering, Zhejiang University, Hangzhou, 310027 People’s Republic of China; 2 ZJU Hangzhou Global Scientific and Technological Innovation Centre; Future Science Research Institute, Zhejiang University, Hangzhou, 311215 People’s Republic of China; 3 Department of Chemistry, Zhejiang University, Hangzhou, 310027 People’s Republic of China; 4 College of Optical Science and Engineering, Zhejiang University (ZJU), Hangzhou, 310058 People’s Republic of China; 5 Faculty of Science, Centre for Clean Energy Technology, School of Mathematical and Physical Sciences, University of Technology Sydney, City Campus, Broadway, NSW 2007 Australia; 6 Institute for Sustainable Transformation, School of Chemical Engineering and Light Industry, Guangdong University of Technology, Guangzhou, 510006 People’s Republic of China

**Keywords:** Sodium metal batteries, Hydrogen-bonded organic framework, Ion-selective transport, Solid electrolyte interphase, Flame-retardant separator

## Abstract

The incessant quest for high-energy-density batteries to meet the growing demand of electrification makes high-level safety operation a critical concern. Traditional polypropylene separators, susceptible to thermal instability and sodium dendrite growth, often lead to internal short circuits and catastrophic thermal runaway. Here, a flame-retardant, dendrite-suppressing, and ion-regulating hydrogen-bonded organic framework (HOF) separator is designed by a simple and scalable strategy. The HOF lattice, featuring abundant polar N–H and C=O sites, preferentially coordinates PF_6_^−^. This selective interaction suppresses anion migration, yielding a high Na^+^ transference number (0.91). Concurrently, the liquid-filled pores and weakly coordinating channels of the HOF facilitate rapid Na^+^ transport, achieving an ionic conductivity of 1.57 mS cm^−1^ at 60 °C. Interfacial analyses reveal that the HOF stabilizes Na^+^ deposition by fostering a NaF-rich solid electrolyte interphase with a high Young’s modulus (~ 11 GPa), which suppresses dendrite penetration. Furthermore, thermogravimetric and combustion tests confirm exceptional resilience above 380 °C and the formation of carbon nitride layer that effectively suppresses heat release. Consequently, Na||Na symmetric cells cycle stably for over 2000 h at 2 mA cm^−2^, while Na||Na_3_V_2_(PO_4_)_3_ full cells retain high capacity ~ 99% over 5000 cycles at 5 C. A pouch cell with a Prussian blue cathode further demonstrates practical applicability with consistent operation at 0.5 C. This multifunctional HOF separator establishes a new paradigm for stable, fast, selective, dendrite-free, and fire-safe sodium metal batteries.

**Supplementary Information:**

The online version contains supplementary material available at 10.1007/s40820-026-02160-5.

## Highlights

- A multifunctional melamine cyanurate hydrogen-bonded framework separator provides intrinsic flame retardancy, high-temperature dimensional stability, and dendrite suppression, offering a practical alternative to commercial polyolefin separators for sodium metal batteries.
- Polar N–H/C=O motifs preferentially interact with PF_6_^−^, suppressing anion migration and boosting Na^+^ transference number, enabling uniform Na^+^ flux and lower polarization.
- Separator-driven interphase regulation forms an inorganic, NaF-rich solid electrolyte interphase confirmed by X-ray photoelectron spectroscopy and time-of-flight secondary ion mass spectrometry 3D mapping, improving Na plating/stripping reversibility and long-term full-cell durability at elevated temperature.

**Supplementary Information:**

The online version contains supplementary material available at 10.1007/s40820-026-02160-5.

## Graphical abstract

**Figure:**
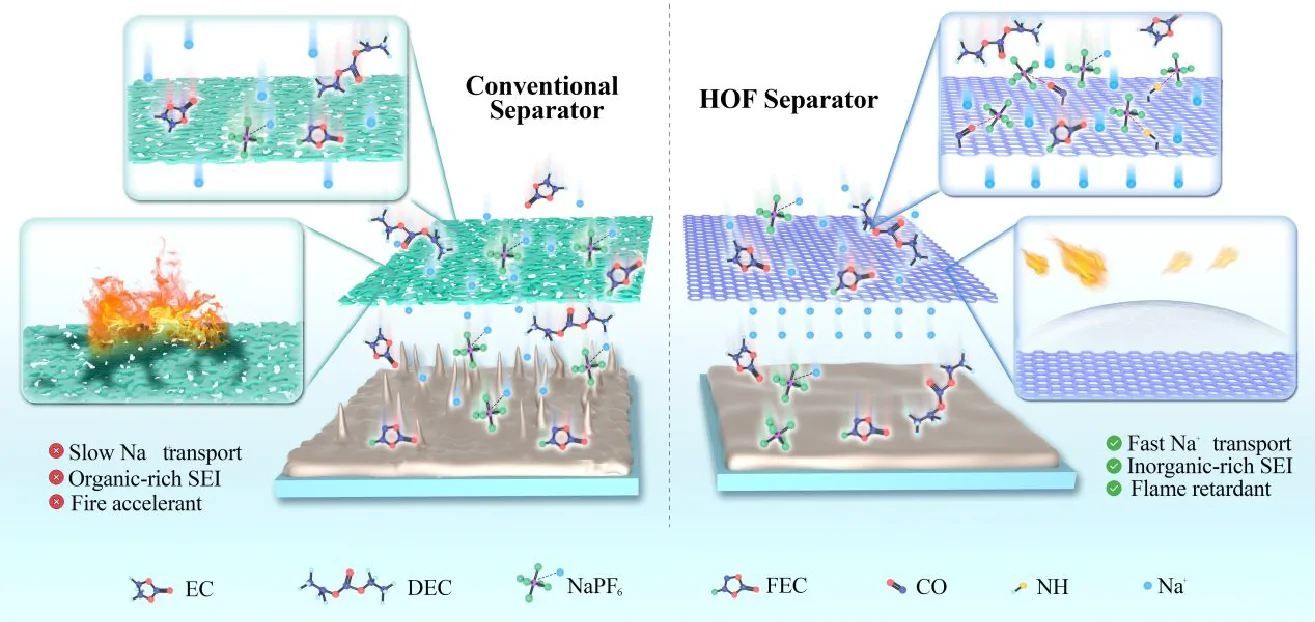

## Introduction

The accelerating demand for sustainable and affordable energy storage technologies has propelled sodium (Na) metal batteries (SMBs) into the spotlight as promising candidates for grid-scale and stationary applications [1–3]. Na offers natural abundance, low cost, and similar electrochemical properties to lithium (Li), while the use of a Na metal anode (SMA) provides an ultrahigh theoretical capacity (1166 mAh g^−1^) and low redox potential (−2.71 V vs. SHE) [4–6]. These attributes render SMBs attractive for large-scale deployment, particularly in regions where Li reserves are limited or unevenly distributed. Despite these advantages, the practical realization of SMBs remains hindered by critical interfacial instabilities, including uncontrollable dendrite growth, incessant solid electrolyte interphase (SEI) breakdown, and electrolyte depletion [7–9]. Together, these phenomena lead to short cycle life, low Coulombic efficiency, severe safety hazards, and thermal runaway. The risk of thermal runaway and battery explosion can be increased in high-energy-density batteries, given that batteries with higher energy density consistently exhibit lower thermal stability during operation. Consequently, ensuring robust safety in SMBs is paramount for the successful development of SMAs.

The separator is an imperative element in battery design, often regarded as an “inactive” component, and plays a decisive role in mediating these challenges. Beyond physically isolating the electrodes, the separator governs electrolyte uptake, ion transport pathways, and interfacial chemistry, which directly dictate dendrite evolution, SEI stability, and thermal safety [10, 11]. Statistical analyses indicate that the failure of the separator, leading to an internal short circuit, is the root cause in approximately 90% of battery failures [12]. Traditional polyolefin separators (such as polypropylene (PP) and polyethylene (PE)) exhibit poor wettability in carbonate-based electrolytes, which compromises Na ion (Na^+^) transport and exacerbates inhomogeneous deposition. Their large, irregular pore structures and lack of ion-selective functionality cannot prevent anion migration, leading to concentration polarization and unstable interphases [13–16]. Most critically, these membranes exhibit low thermal resistance, readily shrinking or melting above 120 °C, increasing the risk of short-circuiting and thermal runaway. Consequently, overcoming the intrinsic limitations of conventional separators is essential to unlocking safe and high-performance SMBs.

To address these shortcomings, various separator engineering strategies have been explored. One approach involves coating polyolefin membranes with inorganic fillers (*e.g.*, Al_2_O_3_, SiO_2_, TiO_2_) [17–19] or flame-retardant additives, which can improve thermal resistance and electrolyte affinity. Another line of research has integrated functional porous materials such as metal-organic frameworks (MOFs) [20] and covalent organic frameworks (COFs) [21] onto separators to regulate ion transport and suppress dendrite growth. While these modifications have demonstrated incremental progress, they often introduce new trade-offs: Inorganic coatings can increase thickness and interfacial resistance, reducing energy density [22]; MOF/COF layers frequently require polymer binders or complex fabrication, limiting mechanical integrity and scalability; and many flame-retardant coatings compromise ionic conductivity [23]. Moreover, the overwhelming reliance on composite designs where a thin functional layer is deposited onto a polyolefin scaffold means the intrinsic thermal vulnerability of the substrate persists, leaving batteries susceptible to catastrophic failure under thermal abuse. Therefore, thin and freestanding separators are a prerequisite that not only withstand high temperature to suppress thermal shrinkage but also are ion-selective with rapid Na^+^ transport and promote uniform deposition of Na in a parallel direction.

**Scheme 1:**
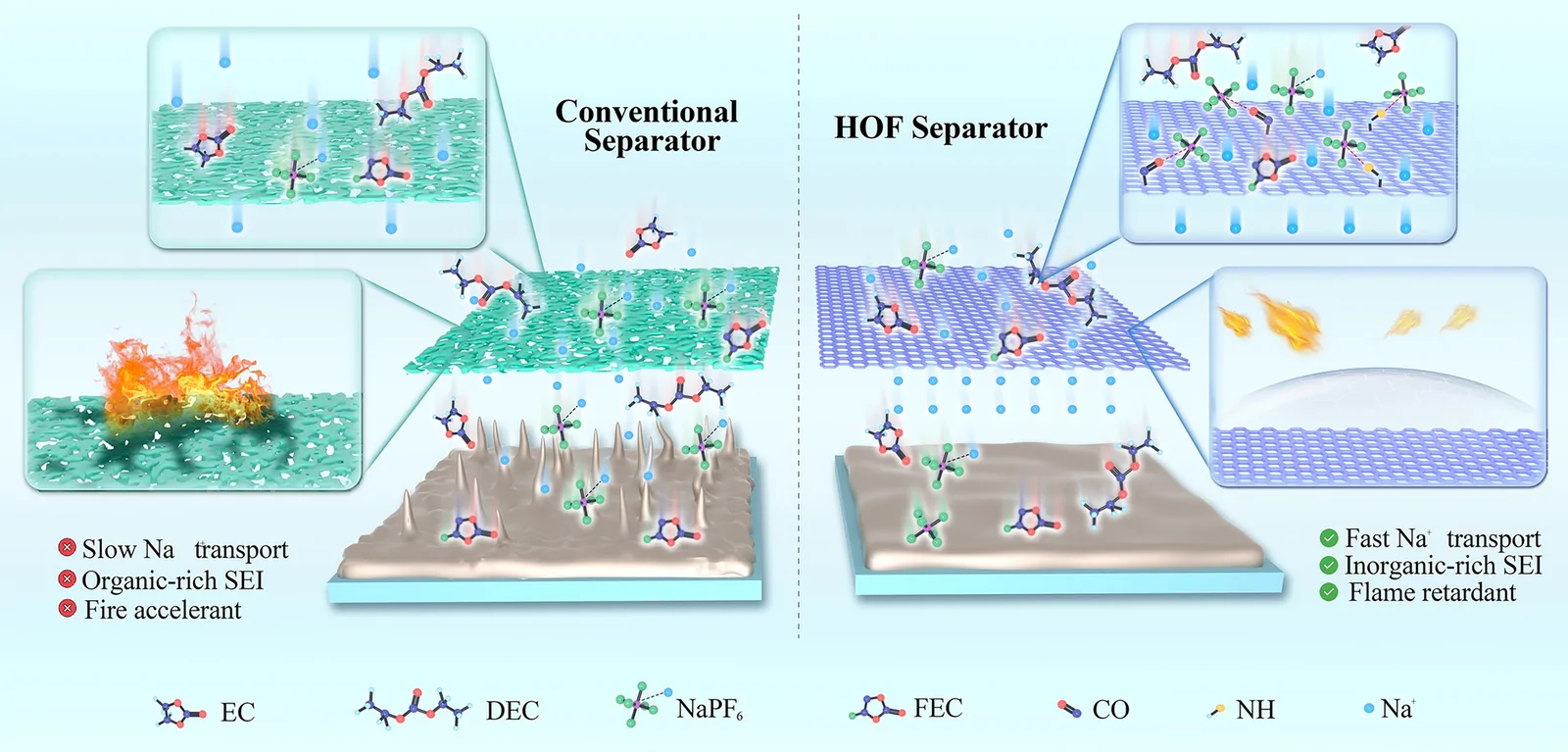
Conceptual illustration of separator design

Commercial PP separators are the most used separator in metal-battery systems, but their lack of ion selectivity and limited thermal safety motivate the development of functional separators, whereas glass fiber separators, despite high porosity, are generally thick, mechanically fragile, and less representative of practical configurations. Here, we report a triazine-based hydrogen-bonded organic framework (HOF) separator, synthesized from melamine cyanurate, as a multifunctional platform for SMBs. Although melamine cyanurate HOFs have been reported and explored as separators in lithium metal batteries, their high-temperature behavior and NaF-rich SEI regulation in SMBs remain unexplored [24]. The crystalline hydrogen-bonded networks, enriched with polar N–H and C=O groups, create well-defined ion-conducting channels that facilitate fast and selective Na⁺ transport while suppressing anion crossover. This ion-regulating behavior promotes the formation of a stable NaF-rich SEI, enhancing interfacial integrity and long-term cycling stability, while the intrinsic flame-retardant properties of the HOF impart superior thermal safety compared with conventional polyolefin membranes (Scheme 1). The supramolecular lattice of the HOF generates abundant polar sites for selective Na⁺ transport, yielding a high Na⁺ transference number (0.91) and fast ionic conductivity (1.57 mS cm^−1^ at 30 μm thickness, 60 °C). These ion-regulating interactions lower the nucleation barrier (107 mV) and homogenize flux, while interfacial analyses reveal that the HOF promotes the formation of a robust, NaF-rich SEI that stabilizes the Na surface. Simultaneously, the framework exhibits outstanding flame-retardant behavior, resisting shrinkage above 380 °C and forming protective carbon nitride layers during combustion. The synergy of ion-selective transport, interfacial engineering, and intrinsic fire resistance enables dendrite-free cycling of Na||Na symmetric cells for over 2000 h and long-term stability of Na||Na_3_V_2_(PO_4_)_3_ full cells 5000 cycles at 5 C under lean electrolyte. The soft-pack pouch cell employing a practical Prussian blue cathode demonstrated stable cycling at 0.5 C with excellent capacity retention. By combining safety and performance within a single supramolecular design, this work establishes HOF separators as a new paradigm for enabling practical SMBs.

## Experimental Section

### Materials

Sodium metal cubes were obtained from Sigma-Aldrich; melamine (99%), cyanuric acid (99%), and polytetrafluoroethylene (PTFE) were purchased from Aladdin Chemistry Co. Ltd. (China); and 1 M NaPF_6_ EC/DEC (1:2) 10%FEC electrolyte was supplied by Duoduo Chemical Technology Co. Ltd (China).

### Material Synthesis

*Synthesis of HOF powder:* HOF powder was synthesized by dissolving equimolar amounts of melamine and cyanuric acid in 100 mL of deionized water under continuous stirring for 30 min. The resulting solution was then aged for 24h at room temperature to promote supramolecular self-assembly. The white precipitate obtained was collected by filtration, thoroughly washed with excess deionized water to remove unreacted monomers, and dried at 60 °C to yield the hydrogen-bonded organic framework (HOF) powder.

*Preparation of HOF separator:* The HOF separator was fabricated by blending HOF powder (90 wt%) with PTFE binder (10 wt%) to form a homogeneous mixture, which was then processed into a gum-like paste. The paste was subsequently hot-rolled to obtain a flexible freestanding membrane, and the thickness was further reduced by repeated rolling, as shown in Fig. S1.

### Electrochemical Measurements

CR2025-type coin cells were assembled in an argon-filled glovebox to evaluate the electrochemical performance of various separators with sodium metal anodes. The electrolyte was 1 M NaPF_6_ in EC/DEC (1:2 by volume) 10% FEC. Sodium foil (12 mm diameter, thickness 45 μm) was used as the counter/reference electrode. For Na||NVP full cells, galvanostatic charge-discharge tests were performed within a voltage window of 2.5-4.0 V, with cathode mass loading between 1.0 and 1.2 mg cm^−2^, and 1 C defined as 120 mAh g^−1^. Na||Cu half cells were cycled between −0.1 and 1 V at a scan rate of 0.1 mV s^−1^. Electrochemical impedance spectroscopy (EIS) over a frequency range of 10^5^ to 10^−3^ Hz with an amplitude of 5 mV, cyclic voltammetry (CV), and linear sweep voltammetry (LSV) were conducted using a CHI760E electrochemical workstation. In situ EIS was performed using a Biologic electrochemical workstation. Readings were taken after every 0.2 V with 15-min rest.

To evaluate the practical applicability of the HOF separator, a single-layer Na||Prussian blue (PB) pouch cell was fabricated. The cathode mass loading was ~ 10 mg cm^−2^, with 1 C defined as 171 mAh g^−1^. The electrolyte was used 7 g Ah^−1^. Thickness of sodium metal was 45 μm. The cell was cycled within a voltage range of 2.5–3.65 V.

### Characterizations

Morphology and elemental distribution were examined by scanning electron microscopy (SEM, JEOL JSM-6360LA) equipped with energy-dispersive X-ray spectroscopy (EDS). X-ray diffraction (XRD) patterns were collected using a Bruker D2 PHASER diffractometer with Cu Kα radiation (*λ* = 1.54056 Å). Nitrogen adsorption–desorption isotherms were measured at 77 K on a Micromeritics analyzer, with specific surface area calculated using the Brunauer–Emmett–Teller (BET) method and pore size distribution derived from density functional theory (DFT) analysis. Contact angles were measured using a DSA100 goniometer to evaluate electrolyte wettability. X-ray photoelectron spectroscopy (XPS, Kratos AXIS Ultra DLD with Al Kα source) was employed to determine elemental valence states.

Operando optical microscopy (Olympus OLS-4000) was used to visualize real-time Na deposition morphology in Na||Na cells. Thermal stability was assessed by thermogravimetric analysis (TGA, Mettler Toledo) and differential scanning calorimetry (DSC, HITACHI STA200), both performed under N_2_ atmosphere at a heating rate of 10 °C min^−1^. Heat release and smoke release properties were evaluated using a microcombustion calorimeter. Time-of-flight secondary ion mass spectrometry (TOF–SIMS, PHI nanoTOF II) was carried out with a sputtering/analysis area of 100 × 100 µm^2^ to probe the depth-resolved SEI composition.

## Results and Discussion

### Synthesis and Physicochemical Properties of HOF Separator

**Fig. 1:**
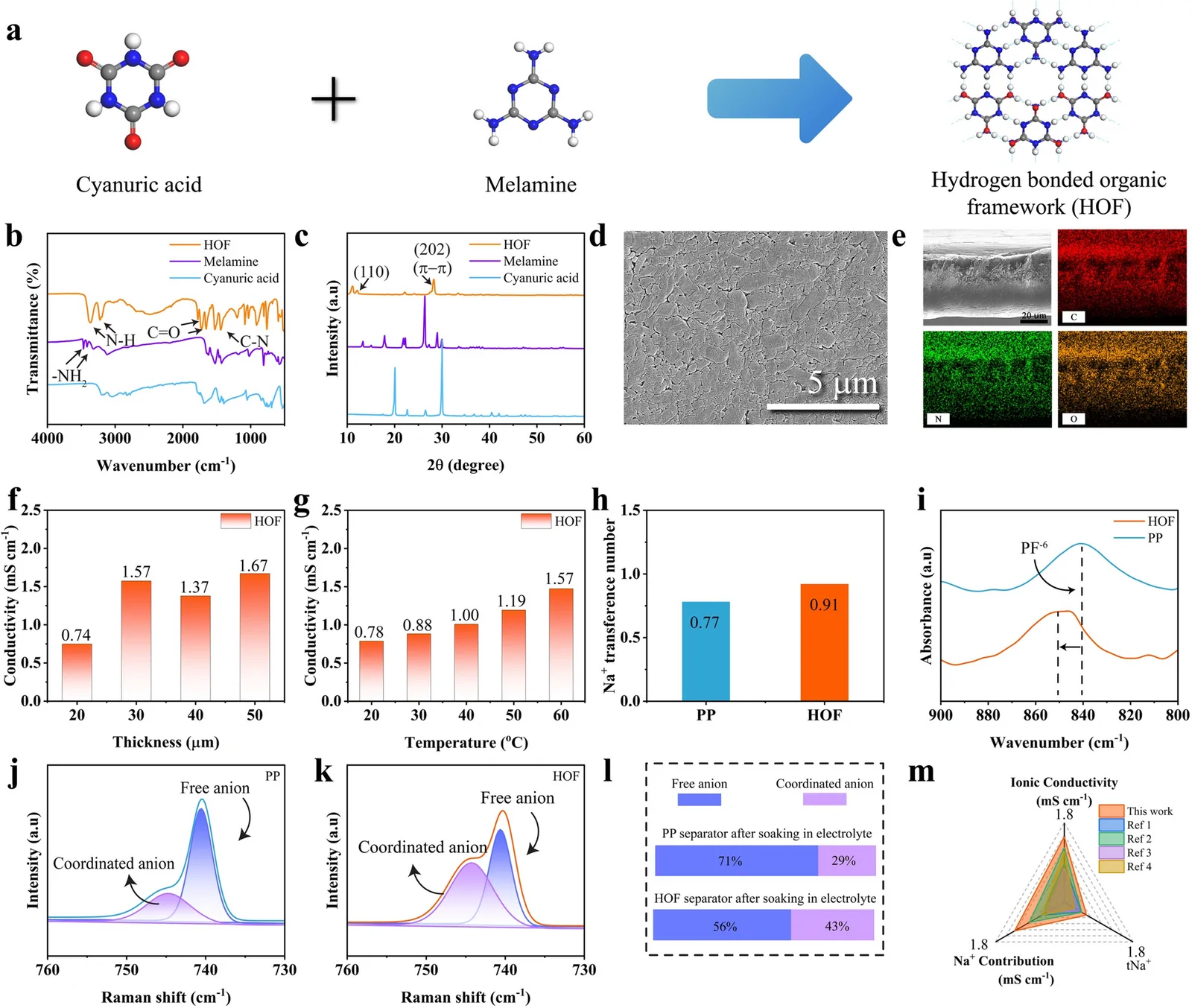
Structural and electrochemical characteristics of the HOF separator. **a** Schematic of the supramolecular self-assembly. **b** FTIR spectra of precursors and HOF. **c** XRD patterns of HOF, melamine, and cyanuric acid. **d, e** SEM cross-sectional view and elemental mapping. **f** Ionic conductivity of HOF separators with different thicknesses. **g** Temperature-dependent ionic conductivity. **h** Na⁺ transference number. **i** ATR–FTIR spectra of PP and HOF separators soaked in electrolyte. Raman spectra of separator soaked in electrolyte **j** PP **k** HOF. **l** Quantitative analysis of PF₆^−^ speciation. **m** Comparison graph

The HOF was synthesized via a facile supramolecular self-assembly of melamine and cyanuric acid, where directional N–H···O hydrogen bonds orchestrated the formation of an ordered, layered architecture (Fig. 1a). Such supramolecular interactions impart structural robustness and generate abundant polar sites capable of coordinating Na⁺, providing the molecular basis for selective ion transport. Fourier-transform infrared spectroscopy (Fig. 1b) revealed shifted and intensified N–H, C=O, and C–N vibrations relative to the pristine precursors, confirming hydrogen-bond formation [25]. These redshift/broadening features are characteristic of N–H···O=C hydrogen bonding and indicate hydrogen-bonded co-assembly, while the long-range order is corroborated by PXRD. X-ray diffraction patterns (Fig. 1c) further evidenced long-range ordering, with characteristic (110) reflections and *π*-*π* associated interlayer stacking (202), which is absent in the monomers [26]; this *π*-*π* associated interlayer stacking was further verified via HR-TEM giving (202) reflection with interlayer spacing 3.3 Å which is consistent with close-packed stacking of *π*-conjugated layers (Fig. S0) [27, 28]. To fabricate a freestanding separator, HOF powders were blended with minimal PTFE binder and hot-rolled into uniform film (Fig. S1), yielding flexible membranes with a dense, defect-free morphology (Fig. 1d, e). Nitrogen sorption analysis confirmed large mesopores and macropore-like textural architecture surface area around 11 m^2^ g^−1^ and pore size with ~ 30–80 nm, which facilitates electrolyte uptake and accelerate ion migration. The non-adsorption behavior of N_2_ in HOFs materials has been similarly reported [29]. The pristine HOF powder consists of plate-like microcrystallites, which can be compacted into a dense membrane during hot-rolling. Consistent with this, contact-angle measurements showed superior wettability for HOF (10°) compared to PP (54°), ensuring rapid electrolyte infiltration and homogeneous ion flux across the membrane (Figs. S2–S5).

To further assess the suitability of the modified separator membrane for SMBs, the ionic conductivity of the membranes was evaluated, which revealed the unique ion transport characteristics of the HOF with ionic conductivity reaching 1.57 mS cm^−1^ at an optimized thickness of 30 μm (Figs. 1f and S6), markedly higher than PP 1.08 mS cm^−1^ while maintaining mechanical integrity. Ionic conductivity increased from 0.78 to 1.57 mS cm^−1^ between 20 and 60 °C (Figs. 1g and S7), indicative of thermally activated Na⁺ migration through the hydrogen-bonded channels. Based on both ionic conductivity and Na∥Na cycling stability, 30 μm was selected. Notably, the Na⁺ transference number was 0.91 in the HOF membrane, which was also substantially higher than PP (0.77) (Figs. 1h and S8), by suppressing anion migration. Linear sweep voltammetry revealed an expanded electrochemical stability window (~ 4.7 V vs. Na/Na⁺) compared to PP (~ 4.2 V), attributable to the robust conjugated network of the HOF framework. Mechanical tensile testing further confirms the robustness of the HOF separator, delivering a higher tensile strength (5.3 MPa) and elongation at break (600%) than the PP separator (4.5 MPa, 411%); consistent with this, the membrane also exhibited excellent flexibility and durability, retaining its integrity under repeated bending, which is critical for practical cell assembly (Figs. S9–S11).

Efficient sodium-ion transport in liquid electrolytes is often impeded by the competitive migration of PF_6_^−^ anions, which lowers the Na^+^ transference number and induces concentration gradients near the separator interface [30]. Conventional PP membranes do little to mitigate this imbalance, permitting random ion flux and exacerbating interfacial instability. By contrast, the HOF separator intrinsically regulates anion behavior through its polar triazine and carbonyl groups, which coordinate PF_6_^−^ anions and promote preferential Na^+^ migration. ATR-FTIR analysis (Fig. 1i) shows that both PP and HOF separators soaked in electrolyte exhibit the characteristic PF_6_^−^ stretching band near 840 cm^−1^; however, the peak is blue-shifted for HOF, indicating stronger electrostatic interactions between PF_6_^−^ anions and the polar sites of the framework [31, 32]. This anchoring effect demonstrates that HOF can effectively immobilize anions, thereby enhancing Na^+^ transport selectivity. Complementary Raman spectroscopy further differentiates free and coordinated PF_6_^−^ species (Fig. 1j, k). Deconvolution of the ~ 740 cm^−1^ [33, 34] band reveals that HOF hosts a substantially higher proportion of coordinated PF_6_^−^ (43%) compared with PP (29%), as summarized in (Fig. 1l). Table S1 shows that the HOF membrane outperforms reported designs in both ionic conductivity and Na⁺ transference number, highlighting its balanced advantage in fast and selective ion transport. Collectively, these results establish that the HOF separator integrates high ionic conductivity, enhanced Na⁺ selectivity, superior wettability, and mechanical robustness within a thermally stable framework. Such multifunctional attributes directly address the shortcomings of polyolefin membranes and lay the foundation for dendrite-free Na deposition, as demonstrated in the following sections.

### Thermal Stability of HOF Separator

**Fig. 2:**
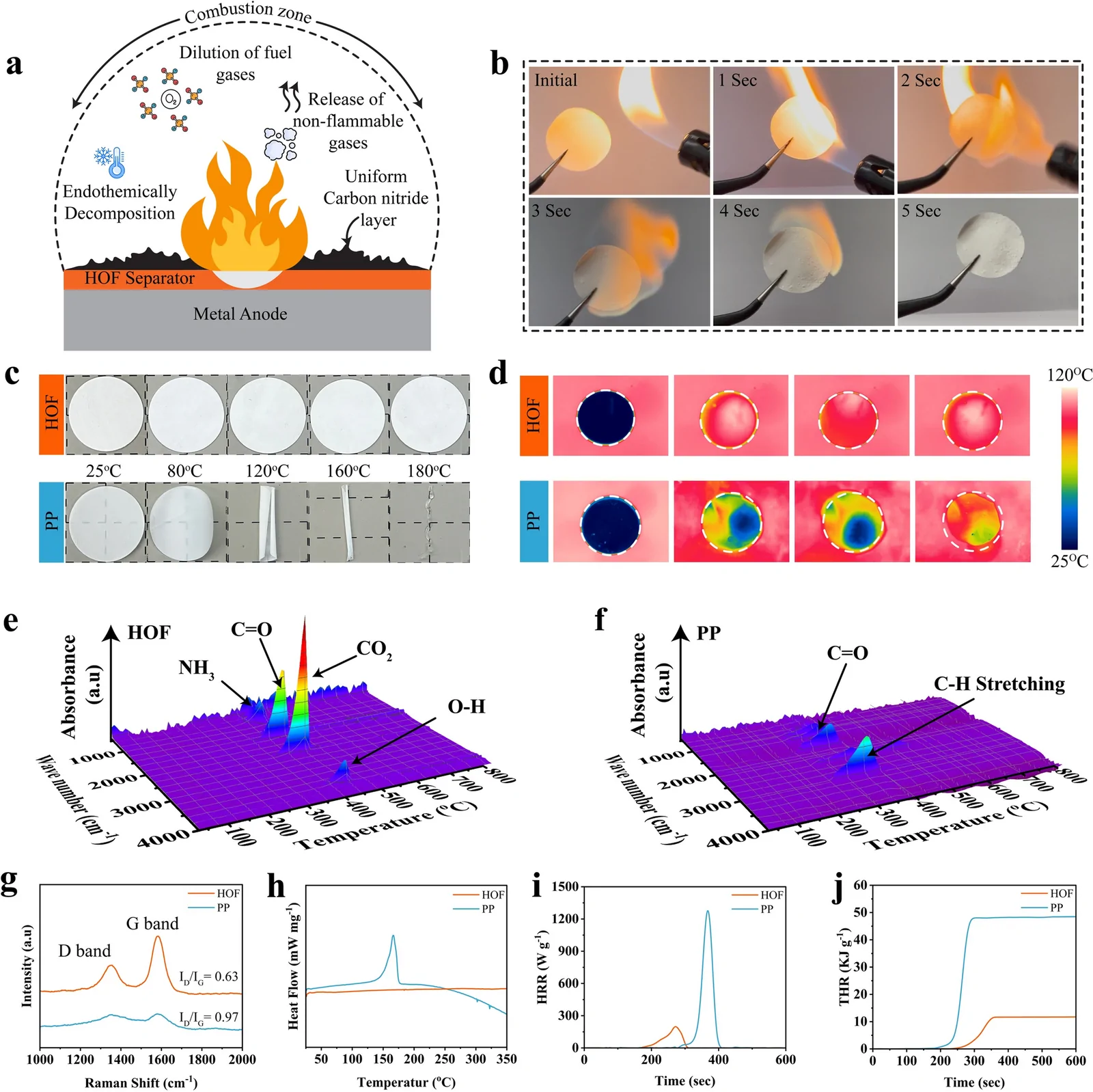
Flame-retardant mechanism and thermal stability of the HOF separator. **a** Schematic illustration of the condensed-phase flame-retardant mechanism of the HOF separator. **b** Time-sequenced combustion images of the HOF separator soaked in electrolyte. **c** High-temperature dimensional stability tests. **d** Thermal imaging of separators under heating.TG-IR of **e** HOF separator and **f** PP separator. **g** Raman spectra of post-combustion residues. **h** Differential scanning calorimetry. **i, j** Microscale combustion calorimetry (HRR, THR)

Separator safety is critical for practical SMBs, where thermal abuse or internal short-circuiting can rapidly trigger catastrophic failure. The HOF separator demonstrates outstanding intrinsic flame-retardant behavior, arising from its hydrogen-bonded supramolecular architecture and condensed-phase conversion to carbon nitride. As illustrated in Fig. 2a, upon exposure to flame, the framework undergoes endothermic decomposition, releasing nonflammable gases (e.g., NH_3_, CO_2_, CO) that dilute the flame while simultaneously forming an in-situ carbon nitride layer that insulates and physically shields the anode. Direct combustion tests (Figs. 2b and S12) reveal that, unlike PP, which shrinks after ignition and melts within seconds, the HOF separator remains structurally intact with a coherent carbon nitride residue and no visible ignition.

To understand the behavior of HOF membranes under elevated temperature, the thermogravimetric analysis (Fig. S13) was carried out, which shows that the HOF separator has a higher thermal decomposition onset (~ 380 °C) than PP (~ 350 °C PP get shrink at 120 °C), indicating superior heat resistance. Notably, HOF retains ~ 20% mass at 800 °C due to the formation of a carbon nitride barrier, whereas PP is almost fully decomposed with negligible residue. The carbon nitride yield suggests a condensed-phase flame-retardant mechanism, where the hydrogen-bonded framework promotes nitrogen-rich carbonization and suppresses volatile fuel release during degradation. Furthermore, the high-temperature dimensional stability tests (25–180 °C, Fig. 2c) show that HOF retains its original size without shrinkage, while PP deforms severely above 120 °C. Thermal imaging confirms uniform heat distribution across the HOF separator with minimal hotspots, in sharp contrast to PP, which exhibits rapid local heating and collapse (Fig. 2d). 3D TG-FTIR spectra reveal that PP decomposition releases strong absorption bands of hydrocarbons (C–H, ~ 2900–3000 cm^−1^) and carbonyl species (C=O, ~ 1700–1800 cm^−1^), indicating emission of combustible gases that sustain flame propagation (Fig. 2e). In contrast, the HOF separator predominantly evolves nonflammable species such as O–H stretching (~ 3600–3700 cm^−1^) and nitrogen-containing gases (C–N/N–H, ~ 1100–1350 cm^−1^), which dilute oxygen and fuel sources, thereby suppressing combustion (Fig. 2f).

Raman spectra of post-combustion residues (Fig. 2g) confirm a graphitized carbon layer in HOF, with a lower I_D_/I_G_ ratio of 0.63 than PP 0.97, indicating higher graphitization and enhanced thermal shielding. DSC analysis (Figs. 2h and S14) further supports these findings. PP exhibits a sharp melting endotherm at ~ 150 °C, typical of crystalline thermoplastics, followed by decomposition near 480 °C. In contrast, HOF lacks a low-temperature melting peak, reflecting its stable hydrogen-bonded network, and shows a pronounced endothermic event at ~ 430 °C from framework nitrogen-rich carbonization. This delayed decomposition aligns with its high TGA residual mass. Microscale combustion calorimetry (Fig. 2i, j) shows that HOF delivers a substantially lower peak heat release rate (pHRR) and total heat release (THR) than PP, confirming reduced flammability. These results confirm the temperature stability of the HOF separator, which prevents structural collapse and actively suppresses thermal runaway through a condensed-phase flame-retardant mechanism. This dual protection structural robustness at elevated temperatures and intrinsic fire retardancy addresses one of the most critical shortcomings of PP membranes and directly enhances the safety profile of SMBs.

### Anion Regulation Ion Transport Characteristics

**Fig. 3:**
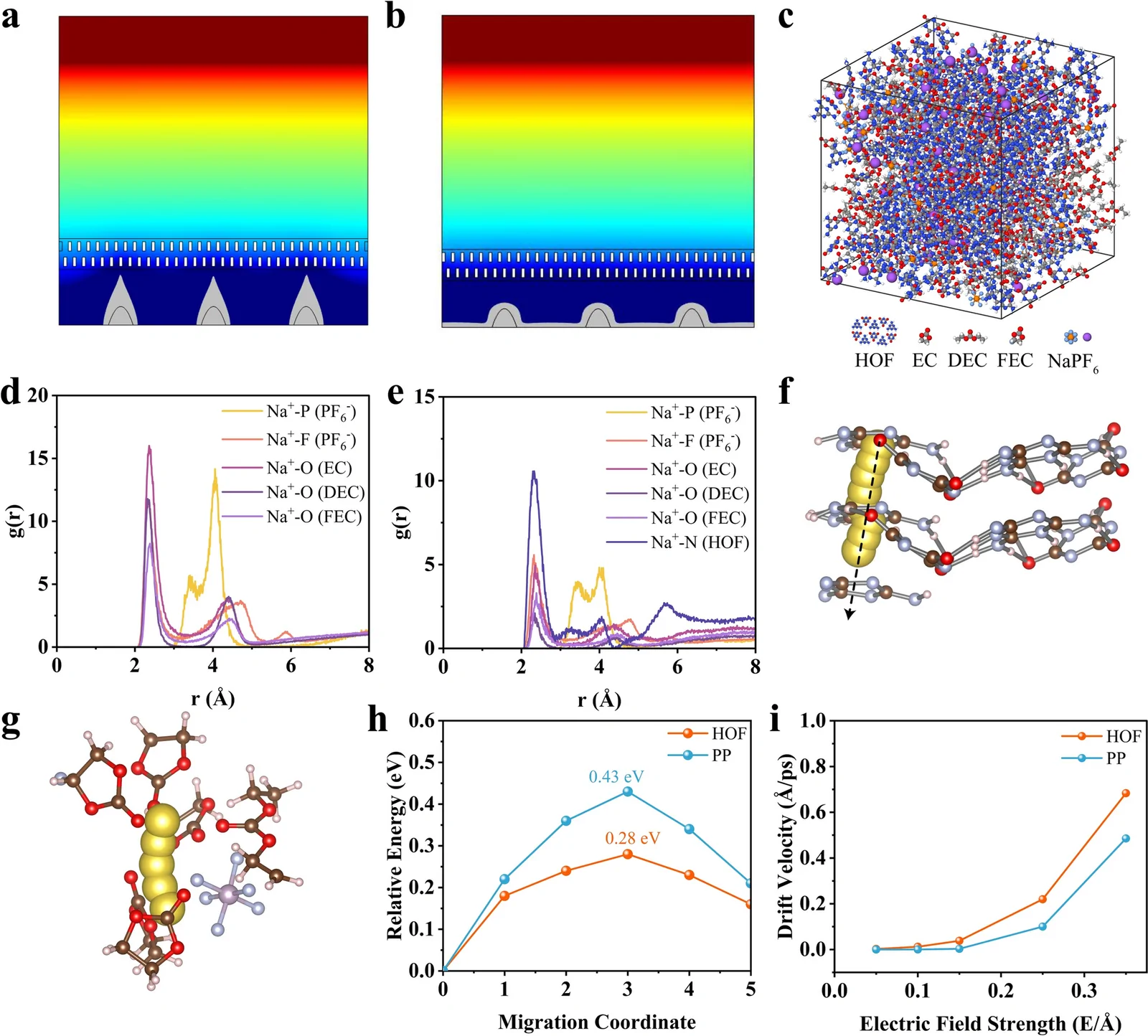
Anion regulation and ion transport characteristics of the HOF separator. Finite-element simulations of Na⁺ flux **a** PP and **b** HOF. **c** Molecular dynamics snapshot. Radial distribution functions (RDFs) **d** electrolyte and **e** HOF in electrolyte. **f, g** Migration pathways. **h** Energy barrier for Na^+^ in PP and HOF. **i** Drift velocity

Theoretical calculations were carried out to further understand the ionic transport of the modified HOF membrane. The finite-element simulations of Na^+^ flux (Figs. 3a, b and S15) reveal that the HOF separator produces a uniform concentration gradient at the electrolyte–anode interface, mitigating localized high-flux “hotspots” that typically initiate dendrite growth. This behavior is consistent with transport theory showing that low cation transference numbers drive concentration gradients and interfacial polarization under load [35]. This effect arises from the immobilization of PF_6_^−^ anions and the continuous Na^+^ transport pathways provided by the ordered HOF structure. Molecular-scale anion regulation and higher t_Na+_ are established strategies to reduce concentration polarization and homogenize interfacial fields [36]. Complementary electric-field and current-density maps (Figs. S16 and S17) further demonstrate that HOF maintains homogeneous interfacial fields over time, whereas PP exhibits localized intensification at surface asperities, confirming the role of the HOF in suppressing uneven ion deposition.

Molecular dynamics simulations provide microscopic insight into this mechanism. Snapshots (Fig. 3c) show that the polar HOF framework offers abundant N and O coordination sites that regulate ion coordination and reshape the local solvation environment without strongly trapping Na^+^. Consistently, DFT binding-energy calculations indicate preferential adsorption of PF_6_^−^ on HOF polar motifs (N–H/C=O) compared to Na^+^ (Fig. S18), supporting anion anchoring and suppressed anion participation in Na^+^ solvation. The electrolyte configuration was optimized in different cubic vacuum boxes (Fig. S19). Radial distribution functions **(**RDFs) support the previous observation in PP, and Na^+^ is predominantly associated with PF_6_^−^ (Na–P and Na–F peaks), indicating strong anion binding (Fig. 3d, e) [37]. In contrast, HOF shifts the coordination preference toward framework heteroatoms (Na–N) and solvent oxygens (Na–O), weakening Na^+^–PF_6_^−^ associations, lowering the desolvation barrier, and facilitating selective Na^+^ transport. The simulated Na^+^ migration pathways (Fig. 3f) confirm this difference in HOF; ions move along continuous conduction channels guided by the framework, while in PP, transport is tortuous and dominated by transient solvent-anion cages (Fig. 3g)**.** Such continuous sub-nanometer channels in COF-type membranes have been experimentally linked to faster and more selective alkali-ion transport [37]. Energy barrier analysis (Fig. 3h) shows that Na^+^ migration requires less activation energy in HOF (0.28 eV) compared to PP (0.43 eV), consistent with easier desolvation and stronger cation-framework interactions. Finally, drift-velocity simulations (Fig. 3i) demonstrate faster and more uniform Na^+^ transport across HOF under increasing electric fields compared to PP. These clearly outline that the HOF separator actively regulates anions at the molecular level, decoupling cation and anion transport. Such a mechanism is crucial for suppressing concentration polarization, stabilizing the interphase, and enabling dendrite-free sodium deposition in subsequent cycling studies. Based on the above structural and physicochemical characterization, the HOF separator not only regulates ion transport but also modifies the Na/electrolyte interfacial chemistry, thereby reducing polarization and promoting stable Na^+^ deposition. Morphologically, the hot-rolled HOF forms a compact, defect-minimized membrane with uniform microstructure, while its polar N–H/C=O sites enable rapid electrolyte wetting/uptake and preferential anion coordination. In addition, it’s improved thermal/flame resistance and mechanical robustness provide a stable separator framework under elevated-temperature operation. To verify this hypothesis, we next quantify the Na anode interfacial kinetics using electrochemical diagnostics.

### Interfacial Stability and Electrochemical Kinetics

**Fig. 4:**
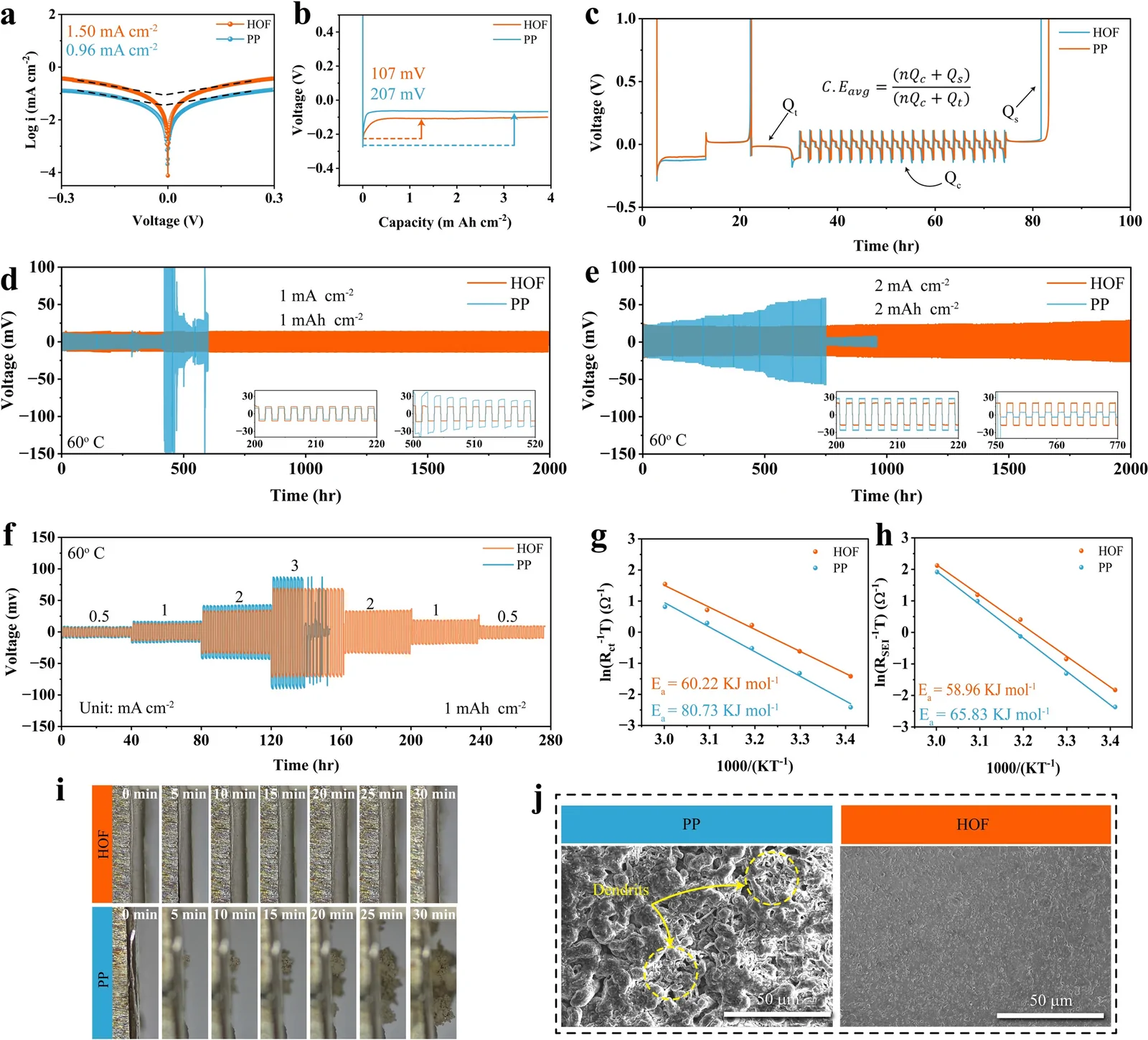
Interfacial stability and electrochemical kinetics. **a** Tafel plots. **b** Nucleation overpotentials of Na deposition. **c** Average Coulombic efficiencies. Long-term cycling of symmetric Na||Na cells at 60 °C. **d** 1 mA cm^−2^ and 1mAh cm^−2^. **e** 2 mA cm^−2^ and 2 mAh cm^−2^. **f** Rate performance tests at 60 °C. Arrhenius plots from EIS. **g** Activation energy of *R*_SEI_. **h** Activation energy of *R*_ct_. **i** In situ optical microscopy. **j** Post-cycling SEM images of Na anodes after 10 cycles at 2 mA cm^−2^ and 2 mAh cm^−2^

Interfacial kinetics at the Na anode are critical in achieving reversible plating and stripping without dendritic growth. A separator that accelerates Na^+^ desolvation and promotes uniform nucleation can significantly reduce interfacial resistance and improve cycling stability. The Na^+^ exchange current density (*j*_0_) and interfacial kinetics were first evaluated by Tafel analysis (Fig. 4a). The HOF separator exhibited a higher j_0_ of 1.50 mA cm^−2^ compared with 0.96 mA cm^−2^ for PP, indicating faster charge-transfer kinetics at the electrode–electrolyte interface. Consistently, the nucleation overpotential (Fig. 4b) was reduced to 107 mV for HOF versus 207 mV for PP, reflecting a lower energy barrier for Na nucleation and more uniform initial deposition. Cyclic voltammetry of Na||Cu cells (Fig. S20) further support these findings. Cells with HOF displayed lower plating potentials and higher deposition currents than those with PP, confirming that the HOF framework enhances electrochemical activity and facilitates charge transport without triggering irreversible side reactions. The average Coulombic efficiency (CE), calculated via Aurbach’s method (Fig. 4c), highlights the advantage of HOF. While PP yielded a CE of only 96.4% due to unstable SEI formation and irreversible Na loss, the HOF separator achieved 99.2%, attributed to the formation of a robust NaF‐rich SEI layer that minimizes dead Na and stabilizes interfacial processes.

Long‐term symmetric cell cycling at 60 and 25 °C highlights the remarkable interfacial stability of the HOF separator. Notably, among the tested HOF thicknesses (20–50 μm), the 30-μm membrane delivers the most stable polarization in Na∥Na cycling and was therefore selected for subsequent evaluations (Fig. S21). The cells tested at room temperature (25 °C) (Fig. S22) exhibit relatively higher overpotentials than those operated at elevated temperature (60 °C) at 1 mA cm^−2^, 1 mAh cm^−2^. Cells equipped with HOF separators operated for over 2000 h at both 1 mA cm^−2^, 1 mAh cm^−2^ and 2 mA cm^−2^, 2 mAh cm^−2^ (60 °C) without short‐circuiting, whereas those with PP suffered from severe voltage fluctuations and premature failure (Fig. 4d, e). A literature comparison of Na∥Na symmetric cell performance is provided in Table S2, highlighting the competitive stability of the HOF separator. Rate performance tests (Fig. 4f) also confirm this robustness. HOF maintained stable voltage responses during abrupt current changes, in contrast to PP, which displayed significant instability and frequent short circuits under the same conditions. Moreover, after 100h of cycling at 1 mA cm^−2^ and 1 mAh cm^−2^, the recovered HOF separator exhibits no obvious visual degradation (Fig. S23), indicating good chemical stability against Na during prolonged cycling.

Arrhenius analysis of temperature‐dependent EIS data (Figs. 4g, h, and S24, S25) provides mechanistic insight into kinetic improvements. Two distinct activation energies were extracted. One corresponds to Na^+^ migration across the SEI (*Eₐ*, *R*_SEI_), and the other is charge transfer (*Eₐ*, *R*_ct_). The *Eₐ*, *R*_SEI_ values were 60.22 kJ mol^−1^ for HOF and 80.73 kJ mol^−1^ for PP, indicating that HOF promotes the formation of a more ion‐permeable and uniform SEI, thereby lowering the energy barrier for Na^+^ transport across the interface. Similarly, the *Eₐ*, *R*_ct_ decreased from 65.83 kJ mol^−1^ with PP to 58.96 kJ mol^−1^ with HOF, suggesting that the polar sites within the HOF framework facilitate efficient solvent-ion dissociation and reduce charge-transfer resistance. These reductions in activation energy collectively enable faster Na^+^ kinetics, improved reversibility, and enhanced high‐rate cycling stability.

The morphological evolution of the Na anode was directly monitored by in situ optical microscopy (Fig. 4i). With the HOF separator, the Na surface remained smooth and dendrite‐free for up to 30 min of plating, whereas dendritic protrusions rapidly formed and propagated in the PP case. Post‐cycling SEM imaging (Fig. 4j) further confirmed uniform, compact Na deposition with HOF, while PP produced loose, dendritic structures prone to short‐circuiting.

Taken together, these results demonstrate that the HOF separator significantly enhances Na^+^ transport kinetics, reduces interfacial resistance, and stabilizes SEI formation, while simultaneously suppressing dendrite growth. These synergistic effects enable highly reversible, long‐term cycling and exceptional stability under high current densities, positioning HOF as a promising separator design for safe and durable SMBs.

**Fig. 5:**
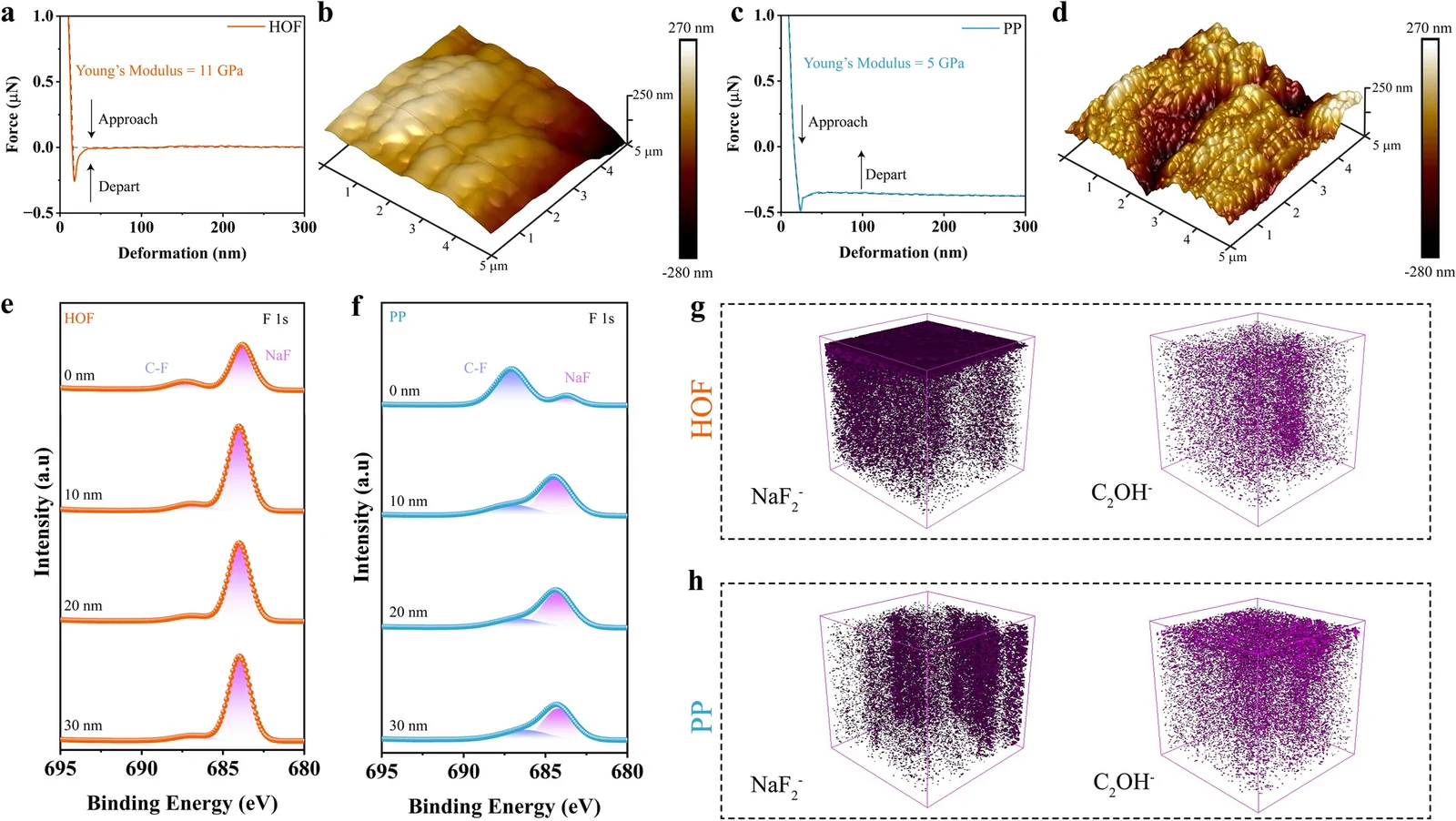
Morphology and chemical composition of SEI layers. AFM force–distance curves of cycled Na anodes with **a** HOF separator and **b** PP separator. 3D AFM surface morphologies of cycled Na anodes with **b** HOF separator and **d** PP separator. Depth-resolved XPS F 1*s* spectra of cycled Na anode with **e** HOF separator and **f** PP separator. TOF–SIMS of Na anode cycled with **g** HOF separator and **h** PP separator

To further understand this excellent electrochemical performance, a detailed analysis of SEI was carried out. The atomic force microscopy (AFM) analysis highlighted the distinct mechanical properties of the SEI layers formed with HOF versus PP separators. Force–distance measurements (Fig. 5a, c) revealed that the SEI derived from HOF exhibits a higher Young’s modulus (~ 11 GPa) compared to that with PP (~ 5 GPa). The enhanced modulus indicates greater interphase stiffness, which is essential for resisting dendritic penetration. Correspondingly, 3D AFM surface topographies (Fig. 5b, d) show that the HOF-derived SEI is compact and smooth, while PP produces a rough and heterogeneous surface, consistent with unstable interfacial chemistry.

The chemical composition of the SEI was further investigated by depth-dependent XPS. Notably, F 1*s* peak-area deconvolution gives a NaF fraction of 86.4% for the HOF-derived SEI vs 16.6% for PP (Figs. 5e, f and S26). For the HOF system, the F 1*s* spectra consistently exhibit a dominant NaF signal from the surface to 30 nm depth, along with minor C–F contributions, confirming the construction of an inorganic-rich, NaF-dominated SEI. Since both HOF and PP were tested under identical FEC-containing electrolyte and cycling conditions, the higher NaF fraction with HOF reflects separator-induced anion regulation rather than an FEC-only effect. In contrast, the PP-derived SEI displays weaker NaF signals and stronger C–F contributions, implying a higher proportion of organic degradation products and a less stable chemical interphase. The C 1*s* profiles reveal that PP-derived SEIs are rich in C–O and C=O functionalities at all depths, reflecting persistent organic electrolyte decomposition, whereas the HOF-protected SEI shows significantly suppressed organic contributions. The Na 1*s* spectra confirm the dominance of NaF in the HOF system, in contrast to weaker and less uniform NaF enrichment with PP (Figs. S27 and S28). The uniform enrichment of NaF in the HOF-protected SEI provides both high ionic conductivity and robust mechanical strength, which contributes to dendrite suppression.

TOF–SIMS profiling further reveals that SEIs formed with PP separators are enriched in organic fragments (C_2_OH^−^, carbonate-related species) distributed throughout the interphase, reflecting continuous electrolyte decomposition. In contrast, the HOF-derived SEI displays dominant inorganic SEI fragments such as NaF^−^ with higher intensity and more uniform distribution across depths. This enrichment in inorganic species, particularly NaF, correlates with the higher mechanical strength observed in AFM and XPS. These analyses demonstrate that the HOF separator facilitates the formation of a NaF-rich, mechanically strong SEI that simultaneously resists dendrite intrusion and ensures efficient ion transport, while the PP separator results in a fragile, organic-rich SEI prone to failure.

### Evaluating Reaction Kinetics and Full-Cell Performance

**Fig. 6:**
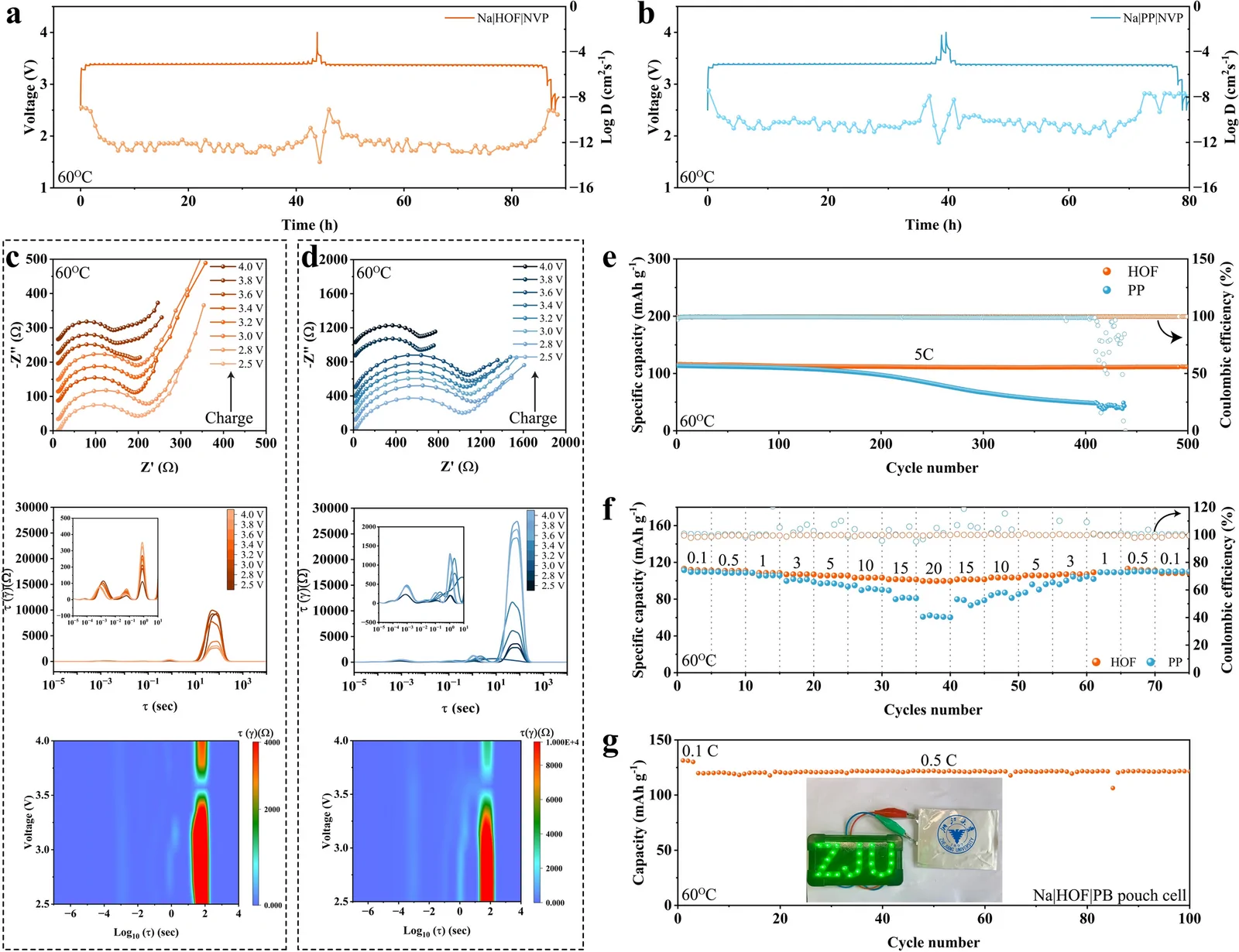
Full-cell electrochemical performance: GITT **a** with HOF separator and **b** with a PP separator. In situ galvanostatic electrochemical impedance spectra (IS-GEIS). **c** Na|HOF|NVP with corresponding DRT analysis and contour mapping at 60 °C. **d** Na|PP|NVP with corresponding DRT analysis and contour mapping. **e** Long-term cycling stability at 60 °C and 5 C. **f** Rate capability. **g** Practical pouch-cell demonstration of Na||HOF||PB

The Na^+^ transport kinetics were first evaluated using the galvanostatic intermittent titration technique (GITT). From the potential response curves (Fig. 6a, b), the Na^+^ diffusion coefficient was calculated to be 2.19 × 10^−12^ cm^2^ s^−1^ for the Na|PP|NVP configuration and 4.08 × 10^−12^ cm^2^ s^−1^ for the Na|HOF|NVP cell. The higher diffusion coefficient obtained with HOF indicates that the hydrogen-bonded framework not only lowers interfacial polarization but also promotes faster Na^+^ migration across the electrode–electrolyte interface. This improved transport ability is consistent with the reduced charge-transfer resistance and smoother Na deposition observed in symmetric cells, establishing the foundation for enhanced full-cell performance.

To monitor the evolution of interfacial processes, in situ galvanostatic electrochemical impedance spectroscopy (IS-GEIS) was conducted at different states of charge. The Nyquist plots for Na|HOF|NVP (Figs. 6c and S29) exhibit nearly overlapping semicircles with minimal variation during extended cycling, confirming stable interfacial kinetics. In sharp contrast, the Na|PP|NVP counterpart (Figs. 6d and S30) shows a progressive increase in impedance and distorted features, indicating interfacial degradation and sluggish ion transport. Distribution of relaxation time (DRT) analysis provides deeper mechanistic insight. For HOF, the spectra remain dominated by small, well-defined peaks corresponding to SEI and charge-transfer resistances, which remain nearly invariant across voltages. This stability confirms reversible charge-transfer processes and suppressed SEI fluctuations. Conversely, PP produces broad and evolving peaks with significantly higher *R*_ct_ values, pointing to unstable SEI chemistry and heterogeneous ion migration.

These interfacial benefits directly translate into superior electrochemical performance. Cyclic voltammetry (Fig. S31) shows sharper and more symmetric redox peaks for Na|HOF|NVP, reflecting faster Na^+^ insertion/extraction kinetics and higher reversibility compared with the broadened and asymmetric peaks in Na|PP|NVP. Likewise, the voltage–capacity profiles recorded at elevated temperature (60 °C) demonstrate the outstanding durability of the HOF-based full cell, which remains stable for over 500 cycles at a high rate of 5 C (Fig. 6e). The corresponding charge–discharge curves (Fig. S32) exhibit highly overlapped and symmetric profiles, indicating minimal polarization, robust interfacial stability, and efficient Na⁺ utilization. In contrast, the PP-based cell shows distorted profiles with progressive polarization and capacity fading (Fig. S33). Notably, even at room temperature, the HOF-based Na|NVP cell delivers excellent long-term stability at 5 C, maintaining nearly constant capacity and Coulombic efficiency over 5000 cycles. In comparison, PP cells fail prematurely, with distorted voltage profiles and pronounced capacity decay within only 500 cycles (Figs. S34–S36).

The rate performance comparison (Fig. 6f) further emphasizes this distinction. The HOF-based cells sustain high reversible capacities across a wide range of current densities from 0.1 to 20 C, highlighting the ability of HOF to homogenize Na^+^ flux and maintain fast ion transport even under severe current stress. By contrast, PP shows rapid capacity fading and unstable efficiencies, underscoring the consequences of uncontrolled dendrite formation and interfacial breakdown. Finally, the scalability of the HOF separator was demonstrated in a soft-packed Na|HOF|PB pouch cell (Fig. 6g). After three initial formation cycles at 0.1 C, the pouch cell exhibited stable operation at 0.5 and consistent capacity retention over extended cycling. This validation confirms that the dendrite-suppressing and SEI-stabilizing effects of HOF are not confined to laboratory coin cells but extend seamlessly to device-relevant pouch configurations, providing a practical pathway toward safer and longer-lasting sodium metal batteries.

## Conclusions

The introduction of HOF separators provides a simple yet powerful route to overcoming the persistent challenges of SMBs. By combining high porosity and electrolyte uptake with robust interfacial chemistry, the HOF separator enables fast Na^+^ transport, lowers activation barriers, and promotes the formation of a stable NaF-rich SEI. These features suppress dendrite growth and interfacial fluctuations, delivering long-term cycling stability at high current densities and elevated temperatures, where conventional PP rapidly fails. Importantly, the benefits extend from symmetric cells to full-cell and soft-pack pouch-cell configurations, where HOF ensures durable capacity retention and high efficiency under practical conditions. This work establishes freestanding HOF separators as a versatile platform for enabling safe, dendrite-free, and high-rate SMBs.

## Supplementary Information

Below is the link to the electronic supplementary material.

Supplementary file1 (DOCX 50637 KB)

## Data Availability

The data that support the findings of this study are available from the corresponding author upon reasonable request.
