# Supplementary material for: A Flame-Retardant Hydrogen-Bonded Organic Framework Separator for Selective Sodium-Ion Transport toward a NaF-Rich Interphase in Sodium Metal Batteries
Source: Nanomicro Lett. 2026 Apr 9;18:325. doi: 10.1007/s40820-026-02160-5 (PMC13066063; doi:10.1007/s40820-026-02160-5)
Supplement: Supplementary file 1 — Supplementary file1 (DOCX 50637 KB) [file 40820_2026_2160_MOESM1_ESM.docx]

Supporting Information for

**A Flame-Retardant Hydrogen-Bonded Organic Framework Separator for Selective Sodium-Ion Transport toward a NaF-Rich Interphase in Sodium Metal Batteries**

Muhammad Ali^1,2^, Moazzam Ali^1,3^, Hamid Hussain^1,2^, Samia Aman^1,2^, Ufra Naseer^4^, Asif Mahmood^5^, Muhammad Tayyab Ahsan^6^, Yinzhu Jiang^1,2,^*, Muhammad Yousaf ^1,2,^*

^1^ School of Materials Science and Engineering, Zhejiang University, Hangzhou 310027, P. R. China

^2^Future Science Research Institute, ZJU Hangzhou Global Scientific and Technological Innovation Centre; Zhejiang University, Hangzhou 311215, P. R. China

^3^ Department of Chemistry, Zhejiang University, Hangzhou, 310027, P. R. China

^4^College of Optical Science and Engineering, Zhejiang University (ZJU), Hangzhou, 310058, P. R. China

^5^Centre for Clean Energy Technology, School of Mathematical and Physical Sciences, Faculty of Science, University of Technology Sydney, City Campus, Broadway, NSW 2007, Australia

^6^Institute for Sustainable Transformation, School of Chemical Engineering and Light Industry, Guangdong University of Technology, Guangzhou 510006, P. R. China

# *Corresponding authors. E-mail: muhammadyousaf@zju.edu.cn (Muhammad Yousaf ); yzjiang@zju.edu.cn (Yinzhu Jiang)

# S1 Finite Element Analysis (FEA)

The simulation used the physical field interface of the thrice current distribution in the electrochemical module of COMSOL Multiphysics software to simulate the deposition of Na^+^ considering various separator. The deposition on the substrate surface mainly includes two main processes:

Mass transport described by the Nernst-Planck equation

$N_{Na^{+}}=-D_{Na^{+}}(\nabla c_{Na^{+}}+\frac{zFc_{Na^{+}}}{RT}\nabla\Phi_{l})$ (S1)

Charge transfer described by Butler–Volmer equation

 (S2)

We performed a 60 s transient simulation of the deposition system with or without HOF separator. The diffusion of ion concentration follows the Nernst-Planck equation, and the reaction on the electrode surface follows the Butler-Volmer equation. To reduce the computational complexity, the actual system is simplified, as shown in **( Fig. S15)**. A model domain with a length of 10 μm and 10 μm is constructed, and a thin film domain with a thickness of 1 μm is constructed at the bottom, and the substrate is Na surface. The top anode region and the boundary on both sides are defined as the un-deformed boundary, and the bottom cathode boundary is set as the deformed electrode surface.The model parameter settings and boundary conditions are shown in Table S1.

# S2 Molecular Dynamics (MD)

Molecular dynamics (MD) simulations were employed to investigate the influence of HOF membrane on Na⁺ ion transport. All simulations were performed using the LAMMPS software package [S1] with a time step of 1 fs. The experimental group was configured in a cubic simulation box with dimensions of 4.6 × 4.6 × 4.6 nm^3^. It contained 50 HOF molecules, 50 molecules each of EC, DEC, and FEC, along with 50 Na^+^ and 50 PF_6_^-^ ions, for a total of 9,050 atoms. For the control system, a cubic simulation box of 4.7 × 4.7 × 4.7 nm^3^ was used, containing 228 molecules each of EC, DEC, and FEC, plus 50 Na⁺ and 50 PF_6_^-^ ions, totaling 9,064 atoms. Periodic boundary conditions were applied in all three dimensions (x, y, z) for both systems.

The OPLS-AA force field [S2] was used to model the ions and molecules. Intermolecular interactions were described by the Lennard-Jones (LJ) and Coulomb potentials, with a cutoff radius of 1.2 nm for the LJ potential. Long-range Coulombic forces were calculated using the Particle-Particle Particle-Mesh (PPPM) method.

Initially, the systems underwent a 1 ns equilibration run in the NVT ensemble at 333.15K K, regulated by a Nosé–Hoover thermostat. Following equilibration, a 10 ns production run was conducted under the same NVT conditions to calculate the Radial Distribution Functions (RDFs). Subsequently, electric fields of varying strengths were applied along the z-direction, and separate 10 ns production runs were performed for each field strength to determine the drift velocity of the Na⁺ ions.

# S3 Density functional theory (DFT)

In Density Functional Theory(DFT) calculations, structural optimizations were performed by Vienna *Ab-initio* Simulation Package(VASP) [S3] with the projector augmented wave (PAW) method [S4]. The exchange-functional was treated using the Perdew-Burke-Ernzerhof (PBE) [S5] functional, in combination with the DFT-D3 correction [S6]. The cut-off energy of the plane-wave basis was set at 450 eV in structural optimizations. For optimization of lattice size of bulk structure of HOF, the Brillouin Zone integration was performed with a Gamma [S7] *k*-point mesh of 4×2×10. For optimization of geometry of electrolyte, the Brillouin Zone integration was performed with a *k*-point mesh of 1×1×1. Since VASP utilizes periodic boundary conditions (PBC) by default, the non-periodic nature of the CEL system was simulated using the fixed cell approach. To minimize spurious interactions between periodic images, the system was placed within a sufficiently large cubic vacuum box. We conducted rigorous convergence tests on the total energy relative to the cell size, evaluating box dimensions of 15, 20, 30, and 35 Å. Our results indicated that the total energy was well-converged and remarkably insensitive to the box volume beyond 15 Å, as shown in Fig Sxxx. Consequently, a cubic box with a side length of 15 Å was selected for all subsequent calculations to balance computational efficiency with physical accuracy. To accurately capture long-range dispersion interactions, which are critical for the geometry of complex molecular systems, the Grimme’s DFT-D3 semi-empirical correction was applied [S8].

The self-consistent calculations applied a convergence energy threshold of 10^-5^ eV. The equilibrium geometries and lattice constants were optimized with maximum stress on each atom within 0.02 eV Å^-1^. Use method climbing image nudged elastic band [S9] to search for the transition state to describe the migration path of sodium ions.

# S4 Ionic conductivity

Ionic conductivity was determined using electrochemical impedance spectroscopy (EIS) on a CHI760E electrochemical workstation, recorded over a frequency range of 10^5^ Hz to 1 mHz with an AC amplitude of 5 mV. For measurements, 60 μL of electrolyte was applied to the separator, which was then sandwiched between stainless steel (SS) blocking electrodes in a CR2025-type coin cell. Ionic conductivity (σ, S cm^-1^) was calculated using Equation (S3):

$\sigma=\frac{L}{RA}$ (S3)

where *R* is the bulk resistance obtained from the high-frequency intercept of the Nyquist plot (Ω), *L* is the separator thickness (cm), and *A* is the electrode area (cm^2^).

# S5 Porosity

The porosity of the separators was determined by liquid uptake using *n*-butanol as the wetting solvent. The porosity (%) was calculated according to Equation (S4):

$Porosity \left( \% \right)= \frac{W_{2}-W_{1}}{V \times\rho}\times100\%$ (S4)

where *W_1_* is the weight of the dry separator, *W_2_* is the weight after immersion in *n*-butanol for 3 h, with surface solvent removed using filter paper, *V* is the geometric volume of the separator, and *ρ* is the density of *n*-butanol.

# S6 Electrolyte uptake

Electrolyte uptake of the separators was determined gravimetrically by recording the weight of the dry separator (*W_1_*) and the weight after soaking in electrolyte for 2 h (*W_2_*). The electrolyte uptake (%) was calculated according to Equation (S5):

$Electrolyte uptake \left( \% \right)= \frac{W_{2}-W_{1}}{W}\times100\%$ (S5)

# S7 Sodium transference number (t_Na+_)

Na||Na symmetric cells were assembled with different separators, and the Na^+^ transference number (*t*Na^+^) at 60^o^C was determined using Equation (S6):

$t_{{Na}^{+}}=\frac{I_{s}(\Delta V-I_{o}R_{o})}{I_{o}(\Delta V-I_{s}R_{s})}$ (S6)

where ΔV is the applied polarization potential (5 mV), *I_0_* and *I_s_* are the initial and steady-state currents, respectively, and *R_0_* and *R_s_* are the corresponding resistances obtained from AC impedance spectroscopy. EIS was recorded using a high-precision electrochemical workstation (Autolab PGSTAT302N) over the frequency range of 10^5^ Hz to 1 mHz to extract resistance values.

# S8 Activation energy

The activation energy (*E_a_*) for Na^+^ transport was determined from temperature-dependent EIS measurements of Na||Na symmetric cells after 10 cycles at 1 mA cm^-2^ and 1 mAh cm^-2^. Impedance spectra were recorded at 293, 303, 313, 323, and 333 K. The activation energy was calculated using the Arrhenius relation (Equation 5):

$\frac{T}{R_{ct}}={Ae}^{\frac{{-E}_{a}}{RT}}$ (S7)

# S9 Columbic Efficiency

The average columbic efficiency was calculated by the given equations. Where n is number of cycles, Q_c_ cycling capacity, Q_t_ first plating capacity, and Q_s_ final stripping capacity.

${CE}_{avg}=\frac{nQ_{c}+ Q_{s}}{nQ_{c}+ Q_{T}}$ (S8)

**Supplementary Figures and Tables**

**
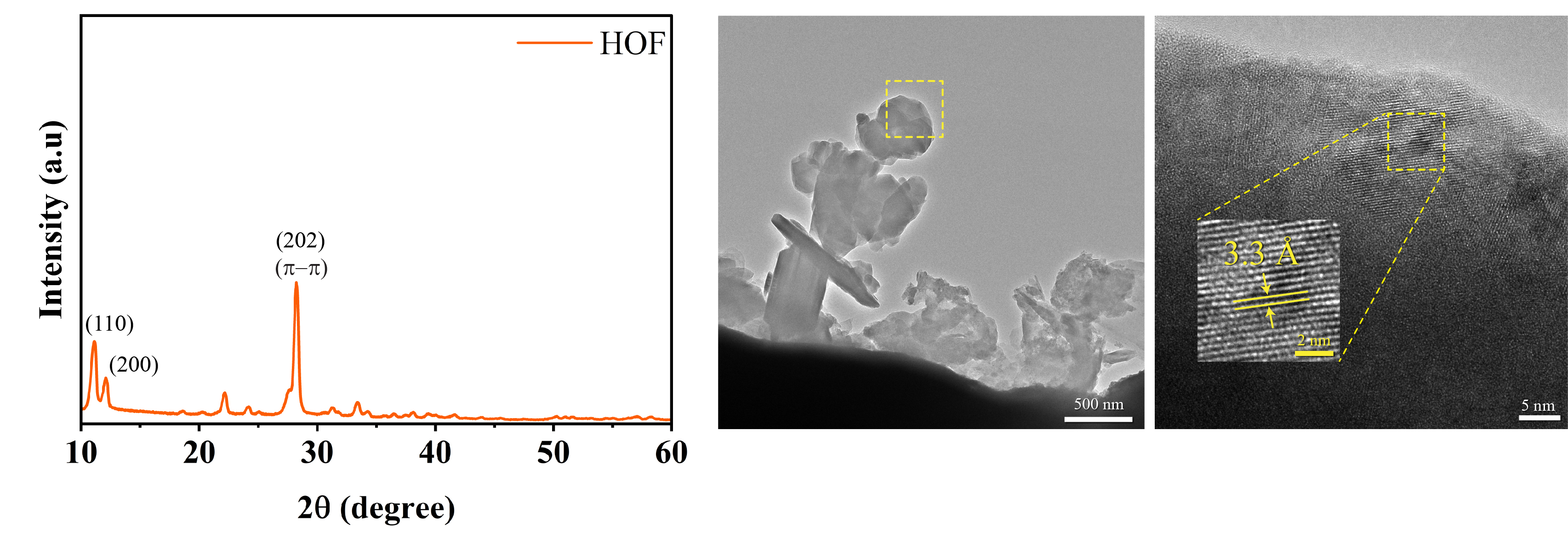
**

**Fig. S0** PXRD pattern and TEM/HRTEM images of the HOF


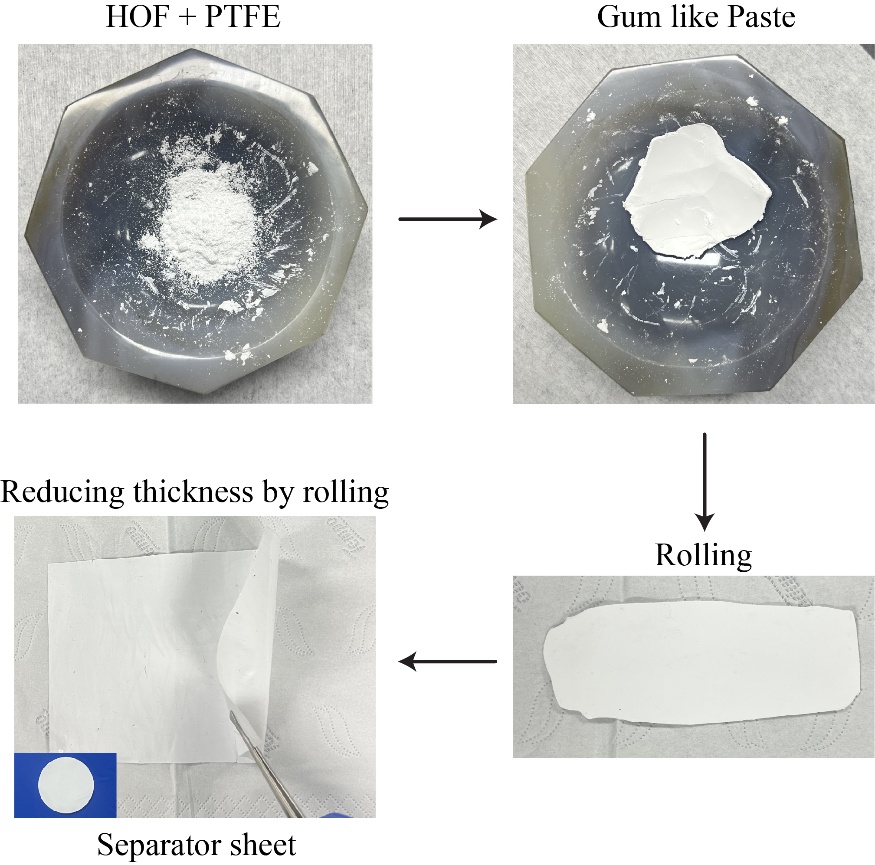


**Fig. S1** Fabrication process of the HOF-based separator sheet


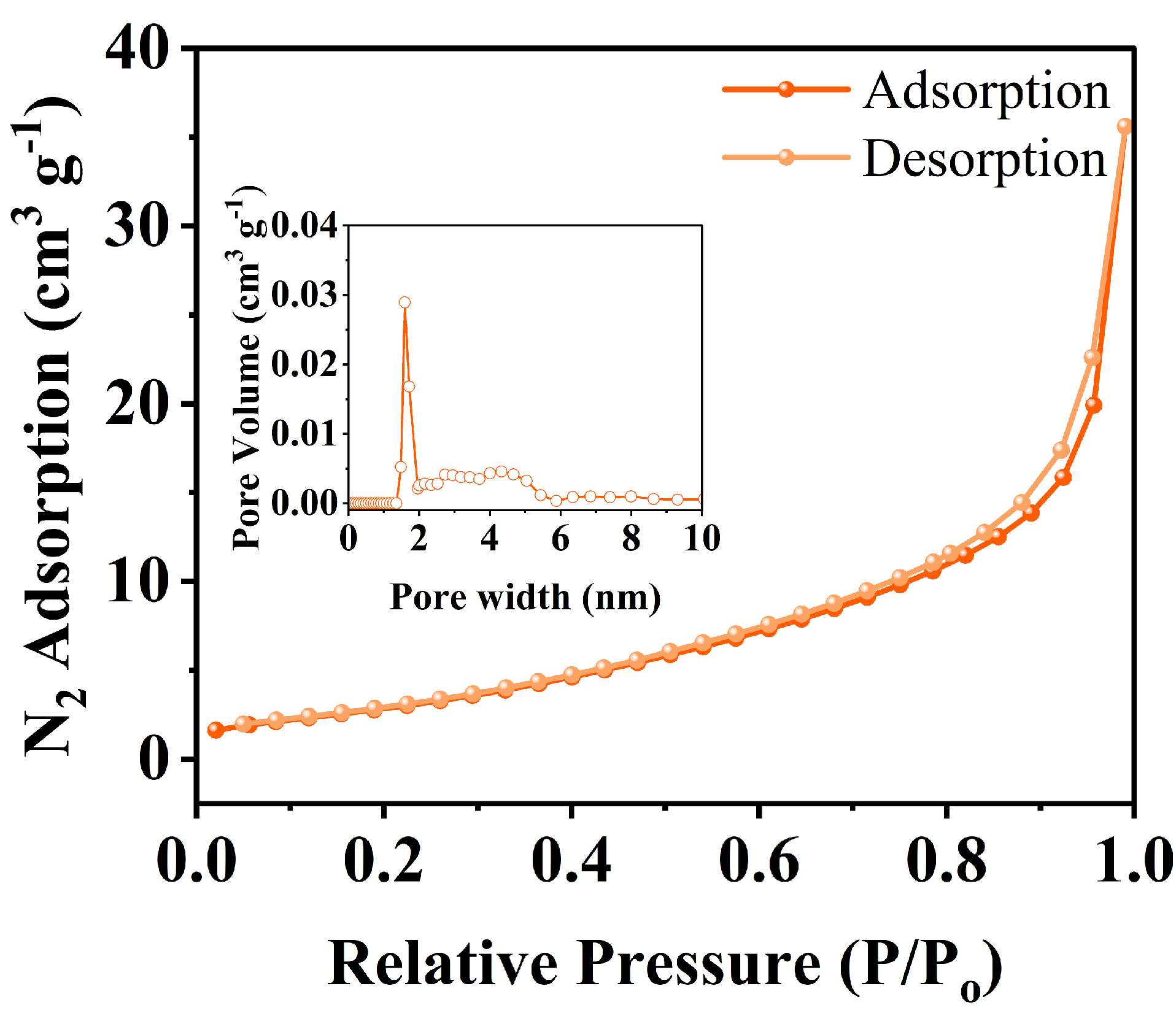


**Fig. S2** N_2_ adsorption–desorption isotherms and pore size distribution


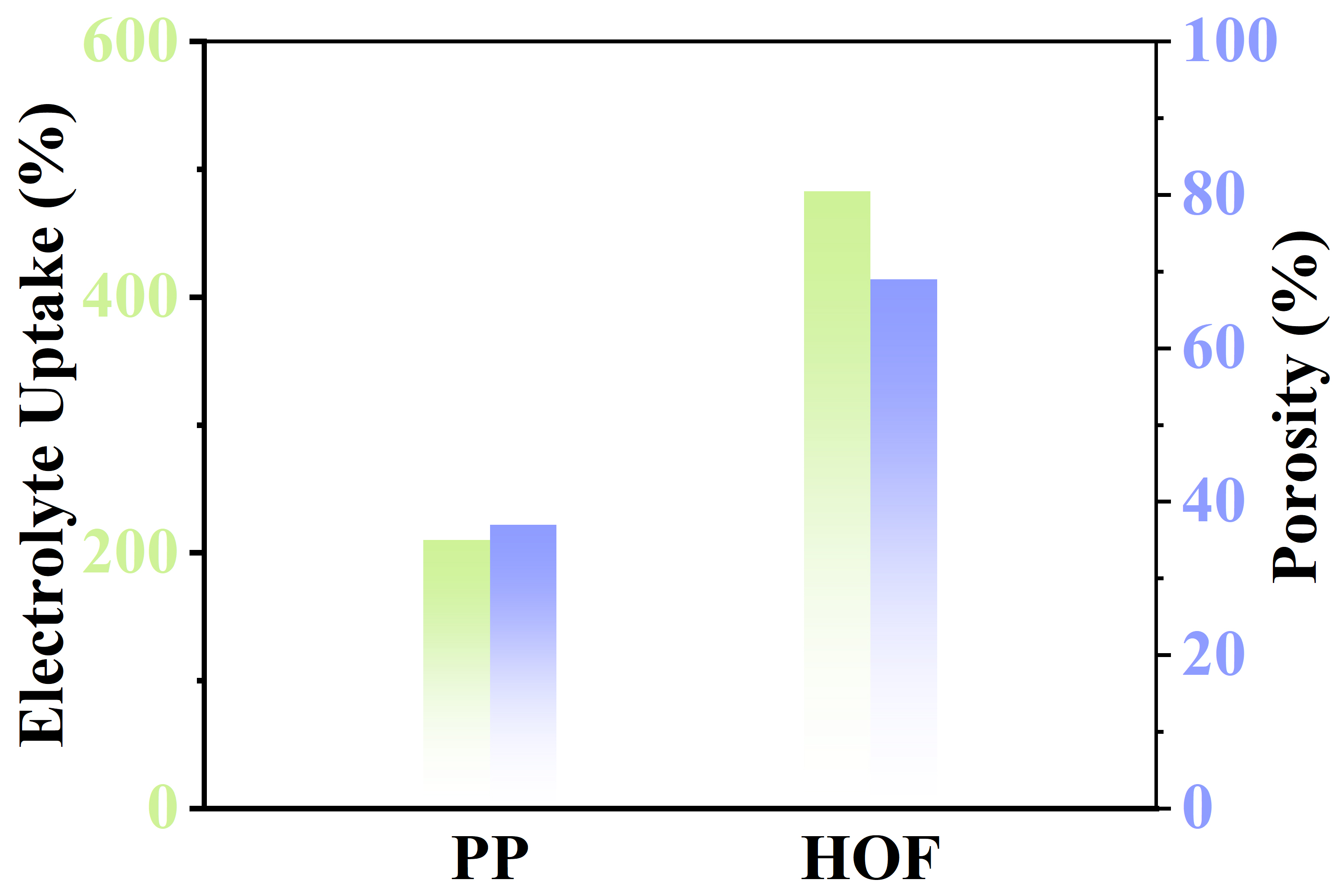


**Fig. S3** Electrolyte uptake and porosity comparison of separators

**
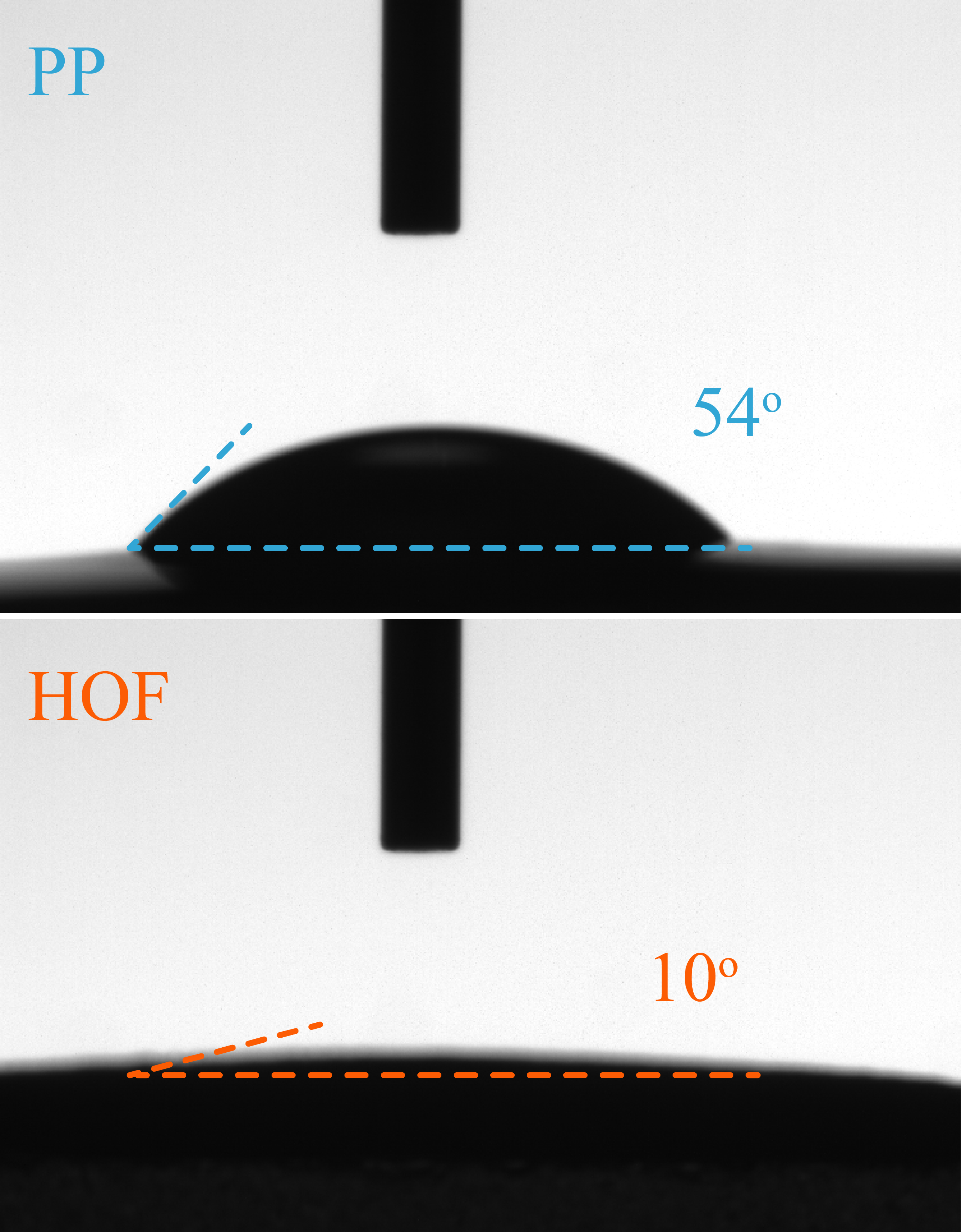
**

**Fig. S4** Contact angle images


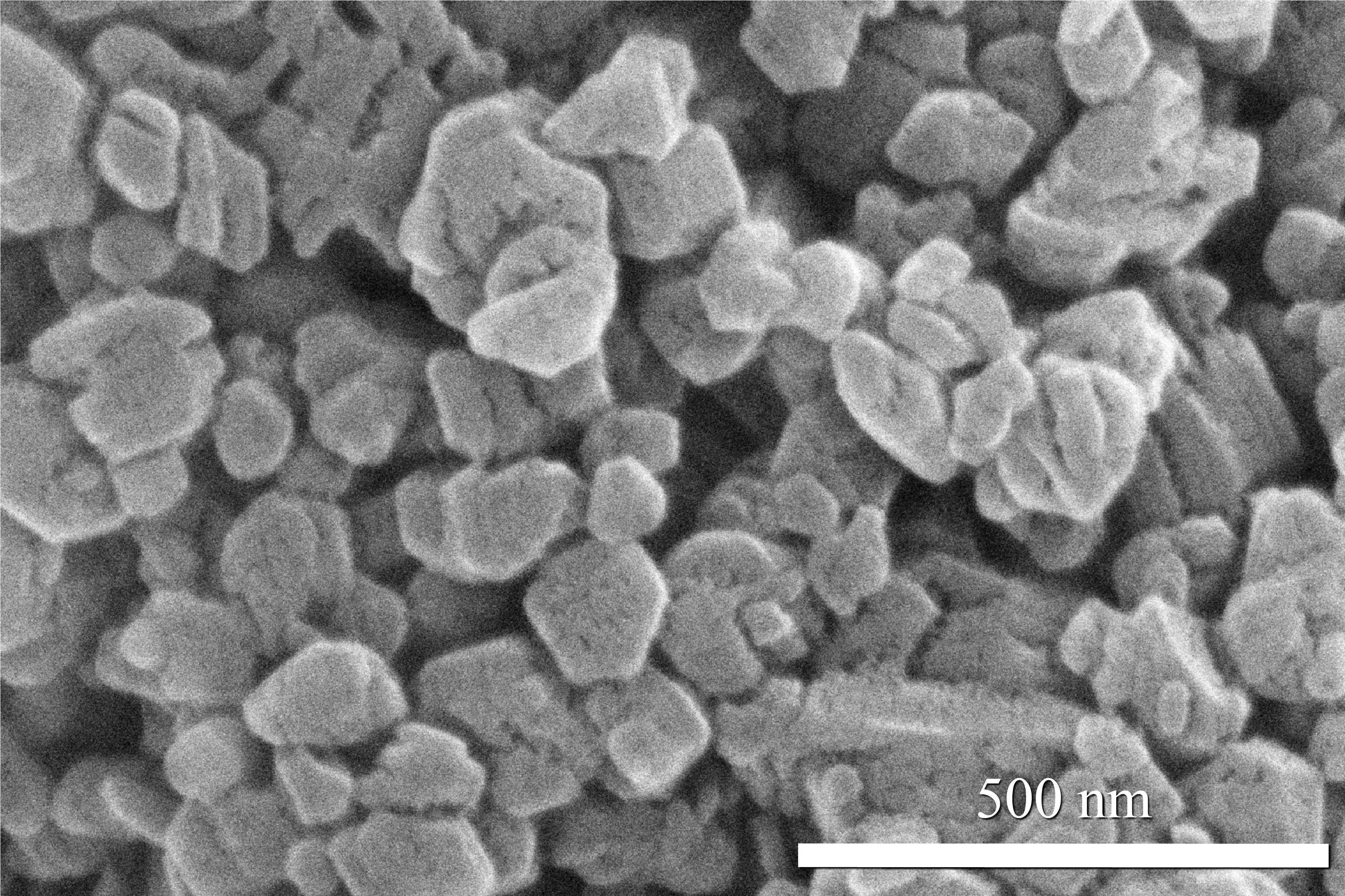


**Fig. S5** SEM image of HOF powder


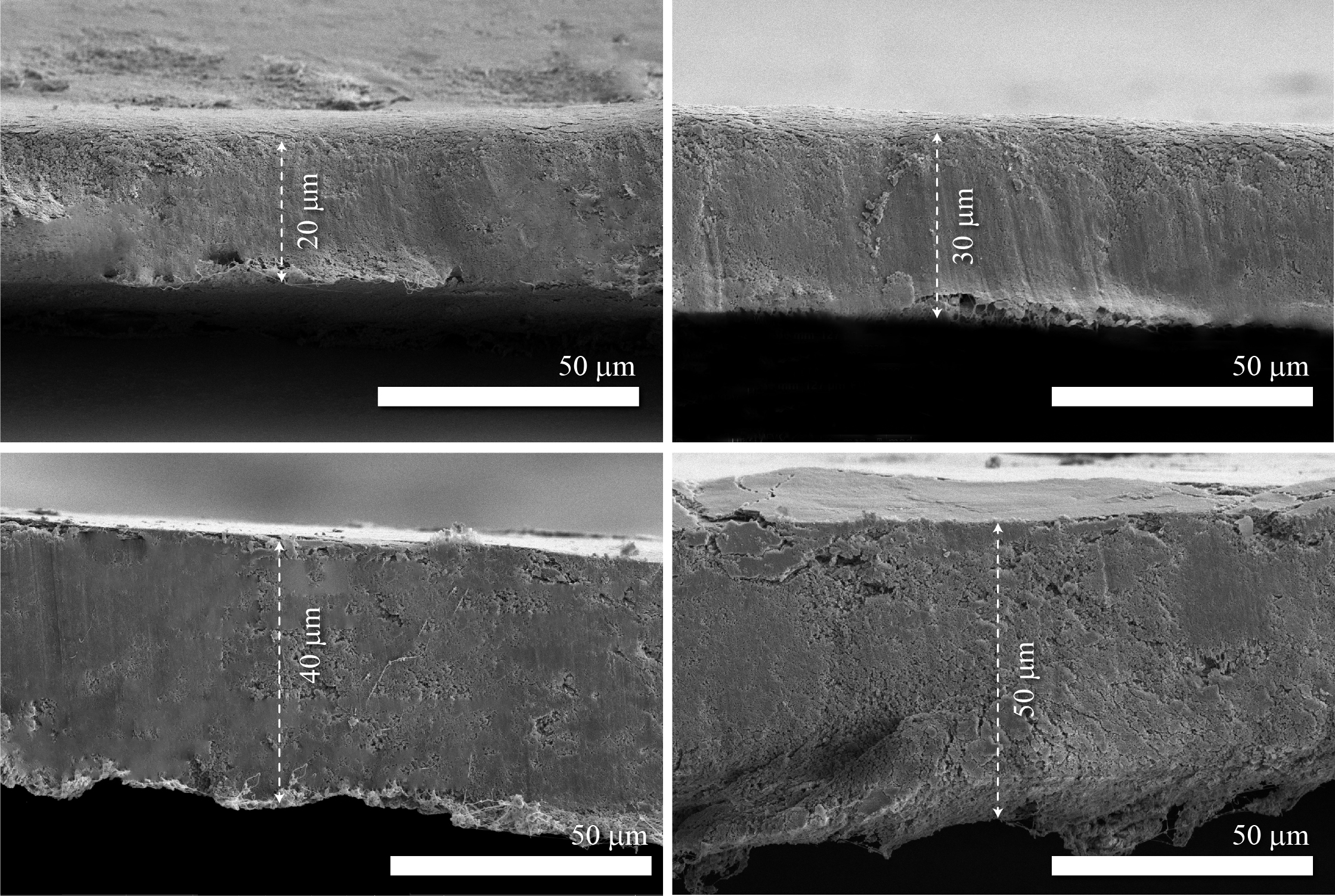


**Fig. S6** Cross-sectional SEM images of HOF separators with different thicknesses


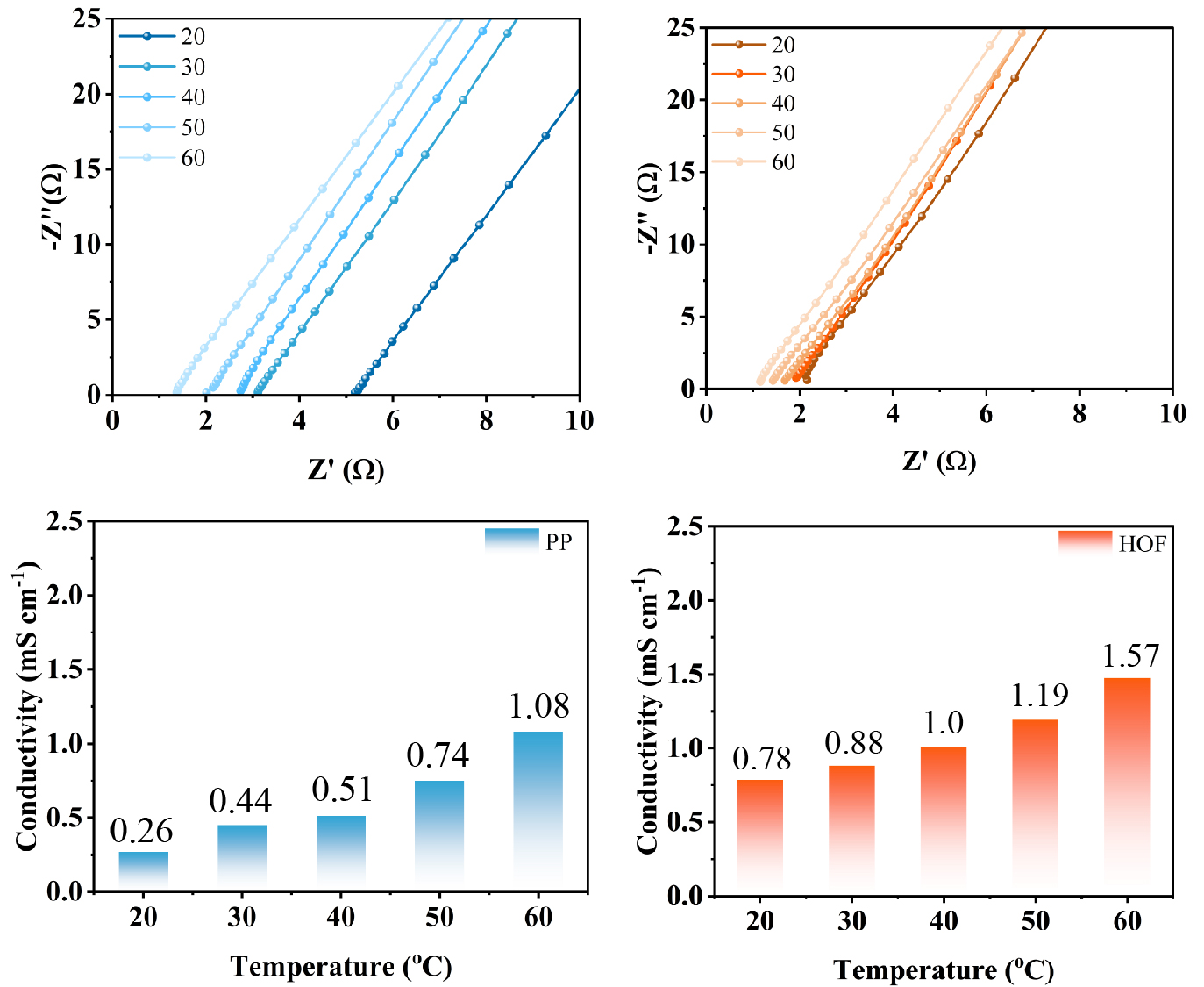


**Fig. S7** Ionic conductivity of PP and HOF separators


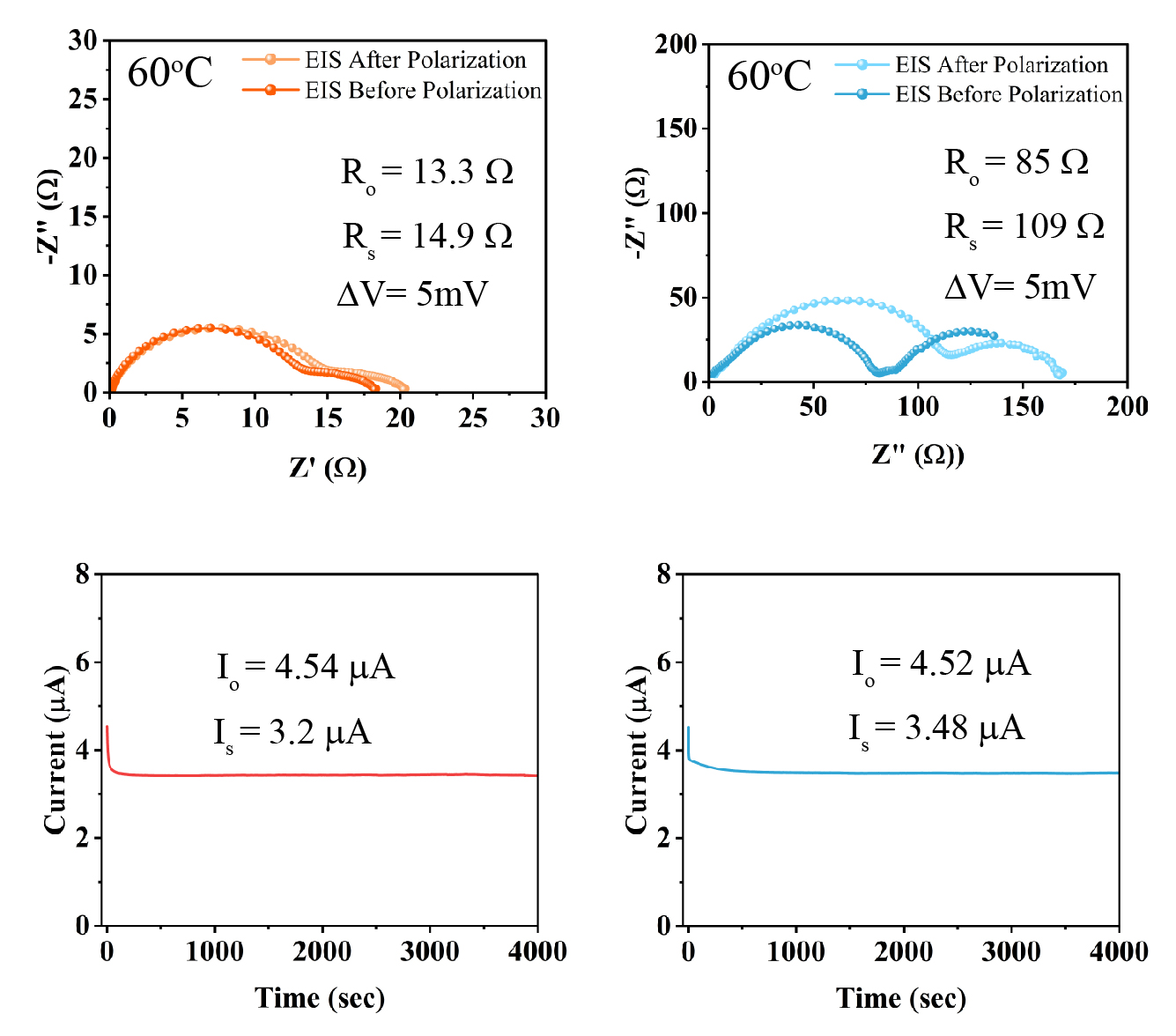


**Fig. S8** Na⁺ transference number of HOF and PP separators


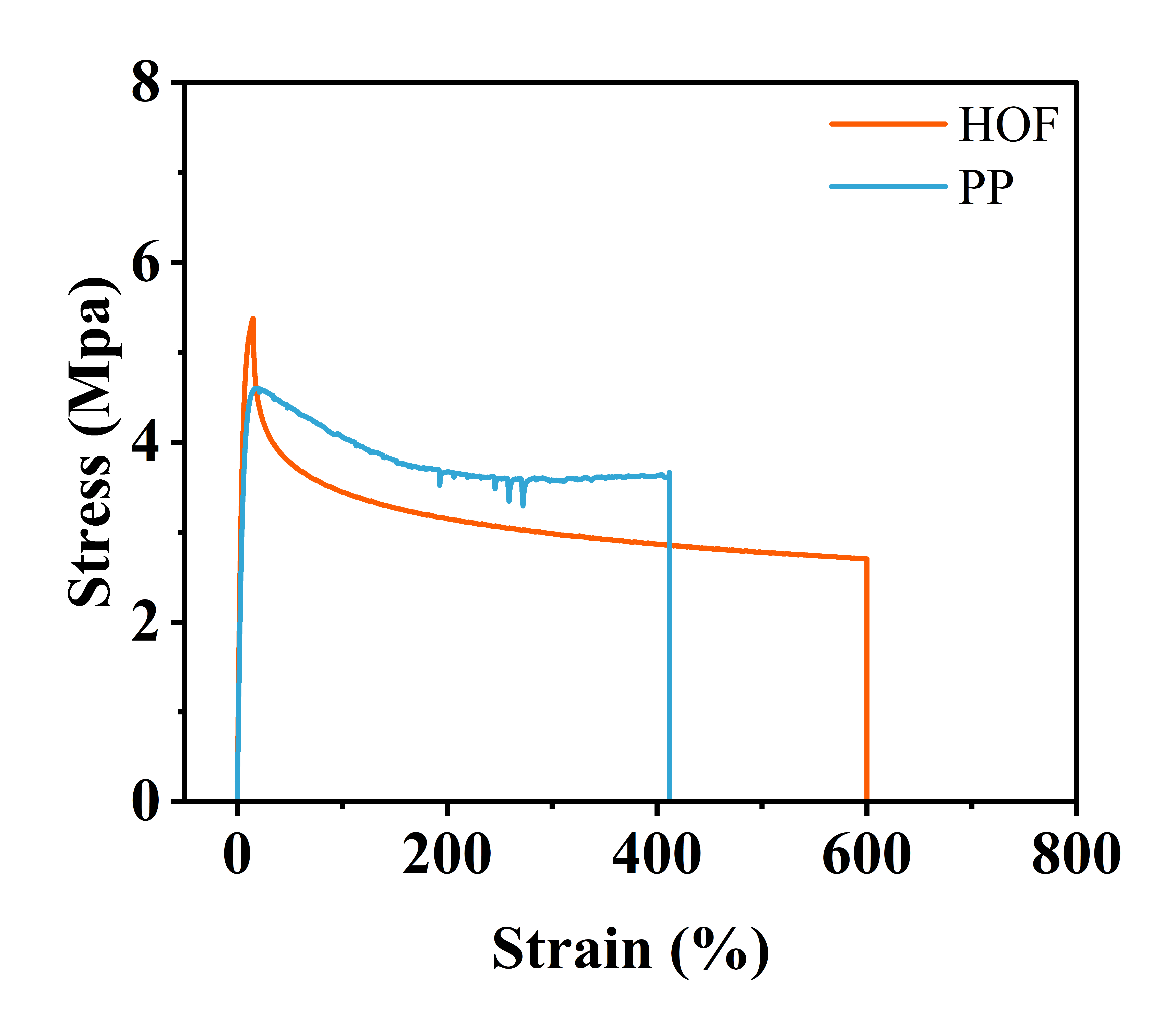


**Fig. S9** Tensile stress–strain curves of the HOF separator and commercial PP separator


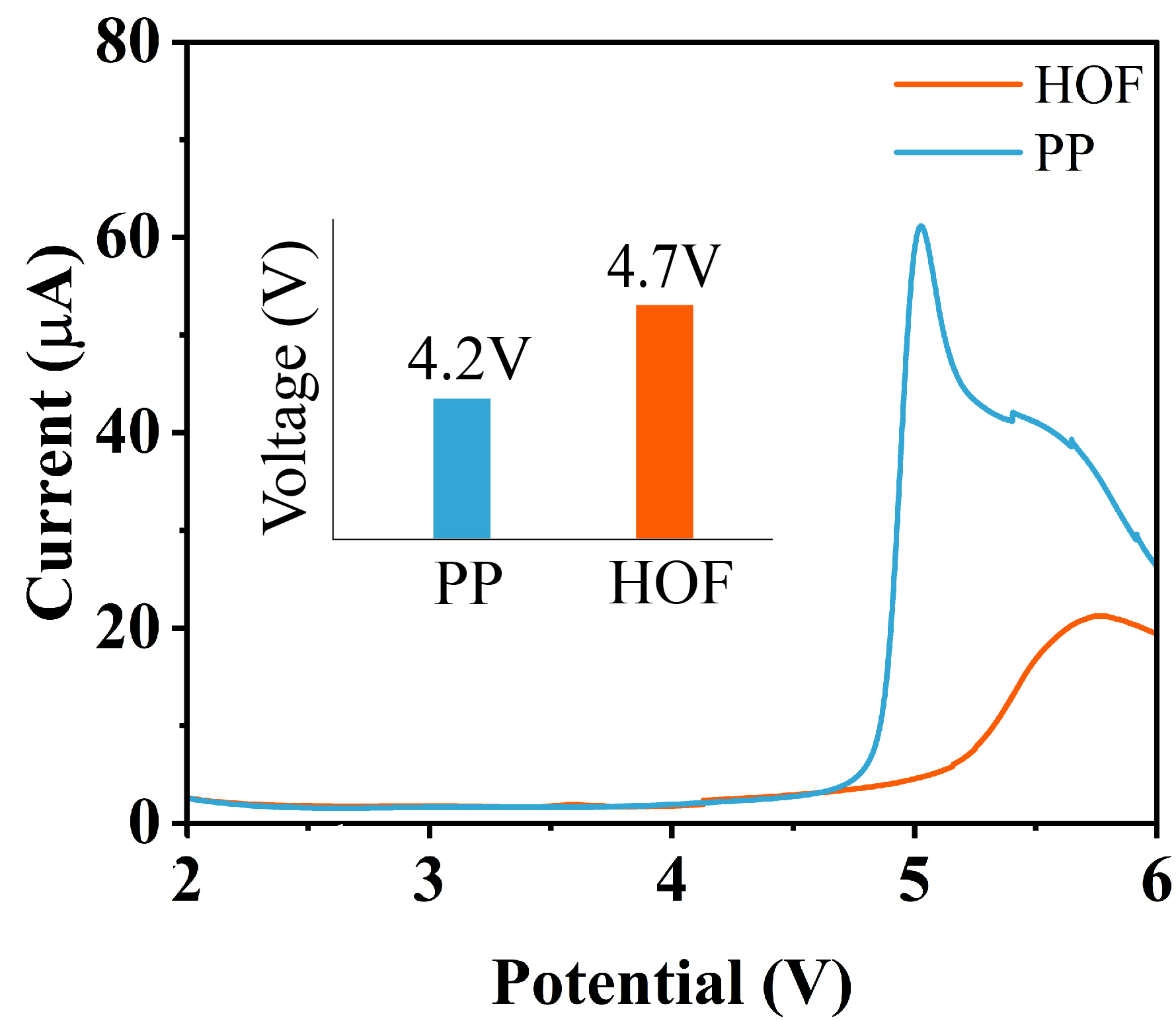


**Fig. S10** Linear sweep voltammetry curves


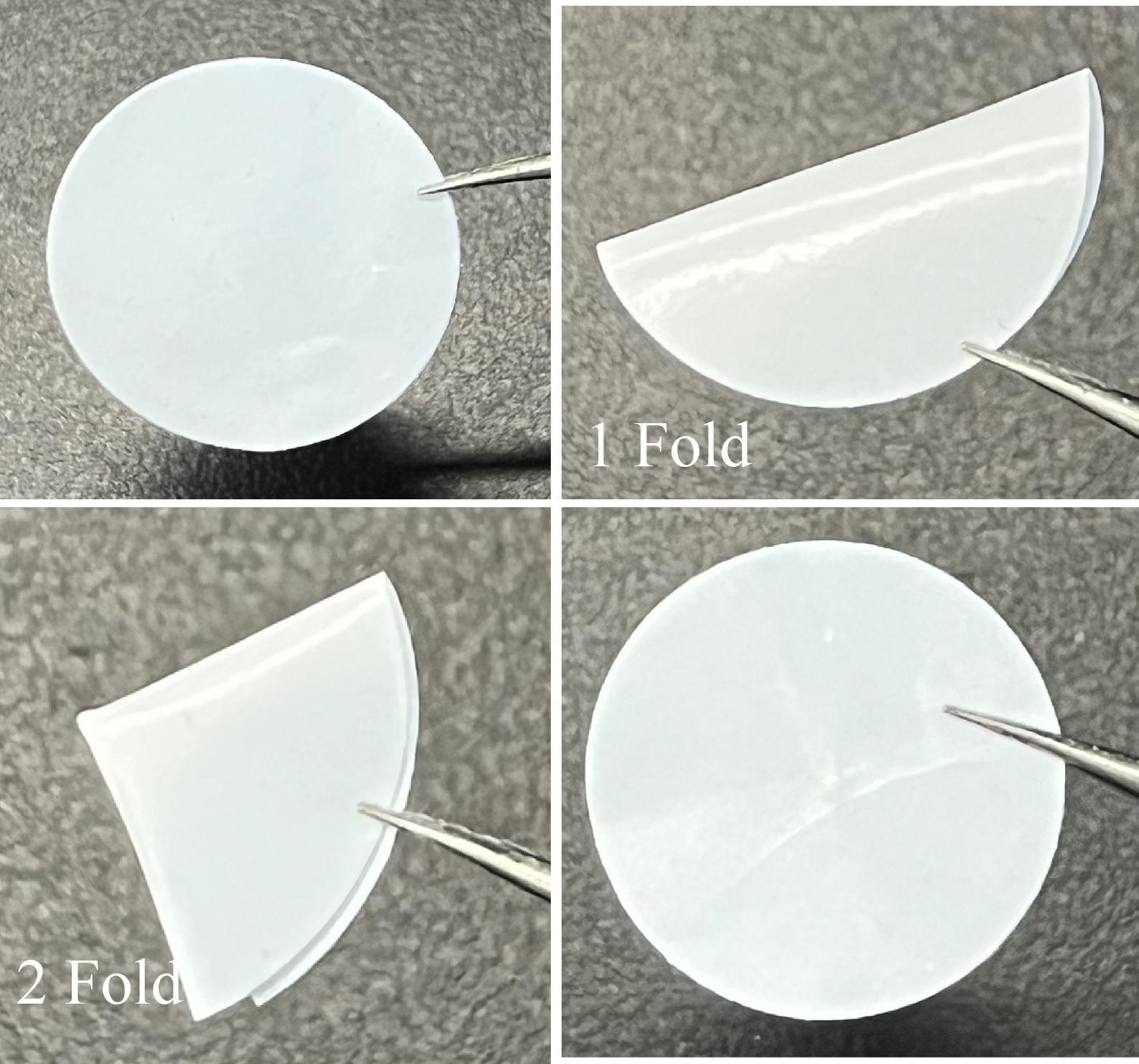


**Fig. S11** Digital photographs of the HOF separator under folding tests

A dry, free-standing HOF separator disk 19mm (diameter) 30um (thickness) was manually folded to 180° and unfolded back to flat for 10 consecutive cycles at room temperature, and photographs were taken after representative folds. No visible cracking, tearing, or delamination was observed after 10 cycles, confirming the mechanical flexibility of the membrane.


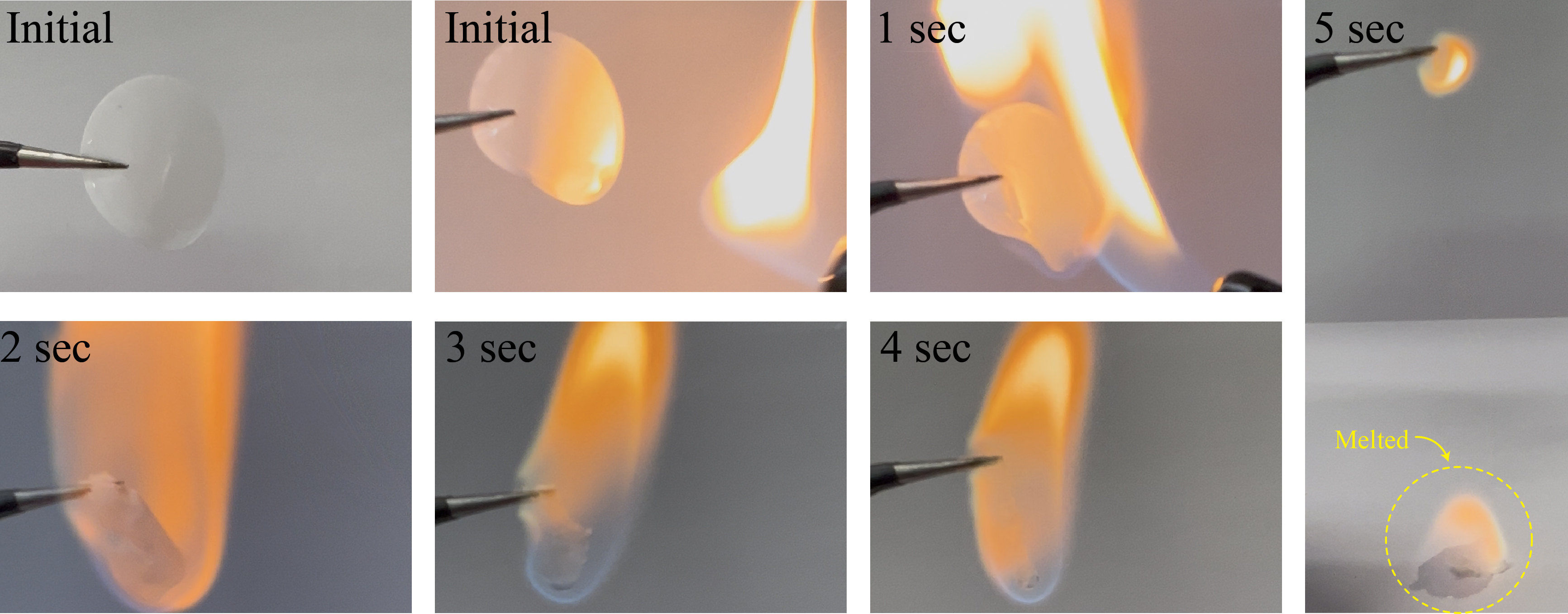


**Fig. S12** Time-sequenced combustion images of PP separator soaked in electrolyte


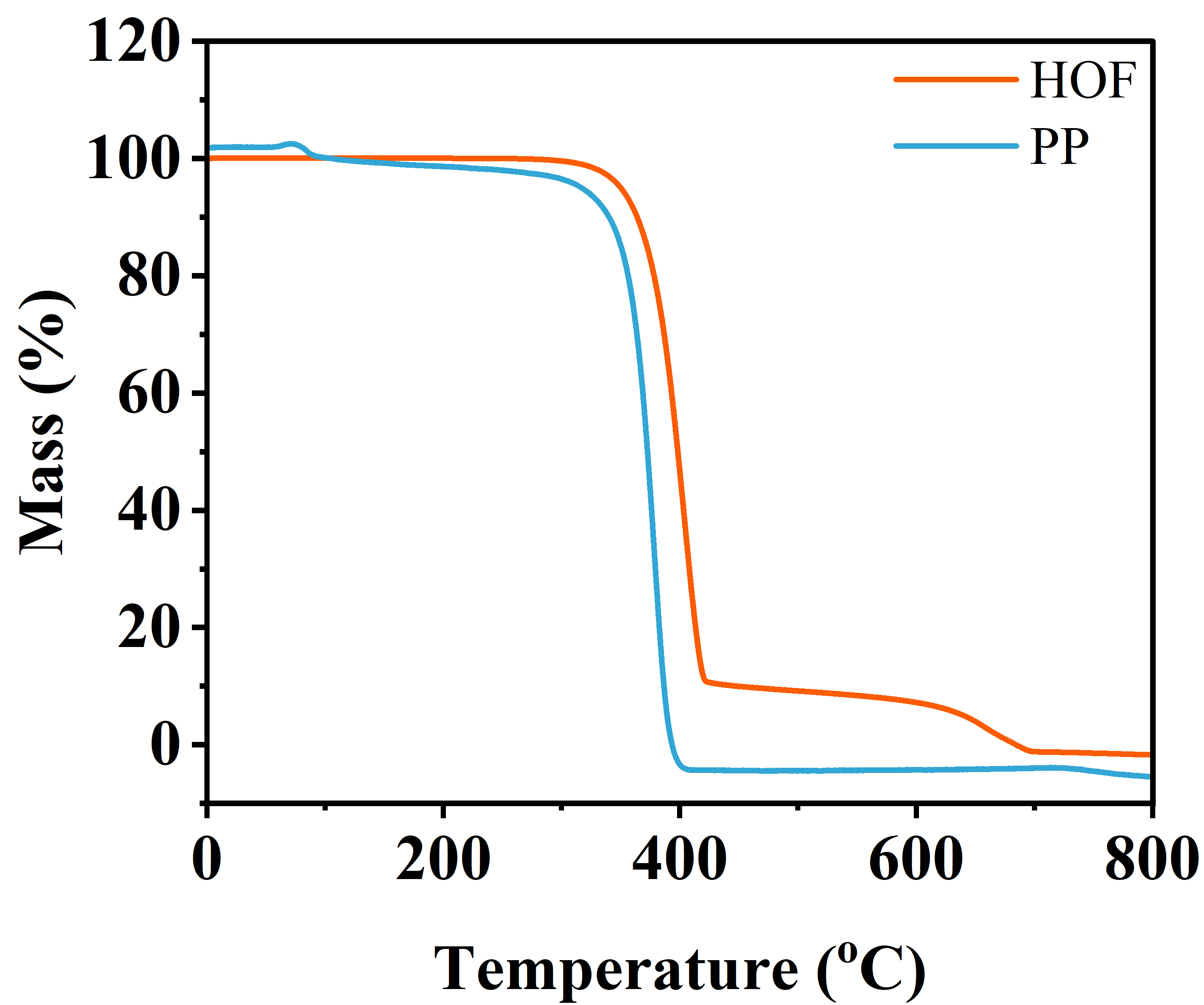


**Fig. S13** Thermogravimetric analysis (TGA) of HOF and PP separators


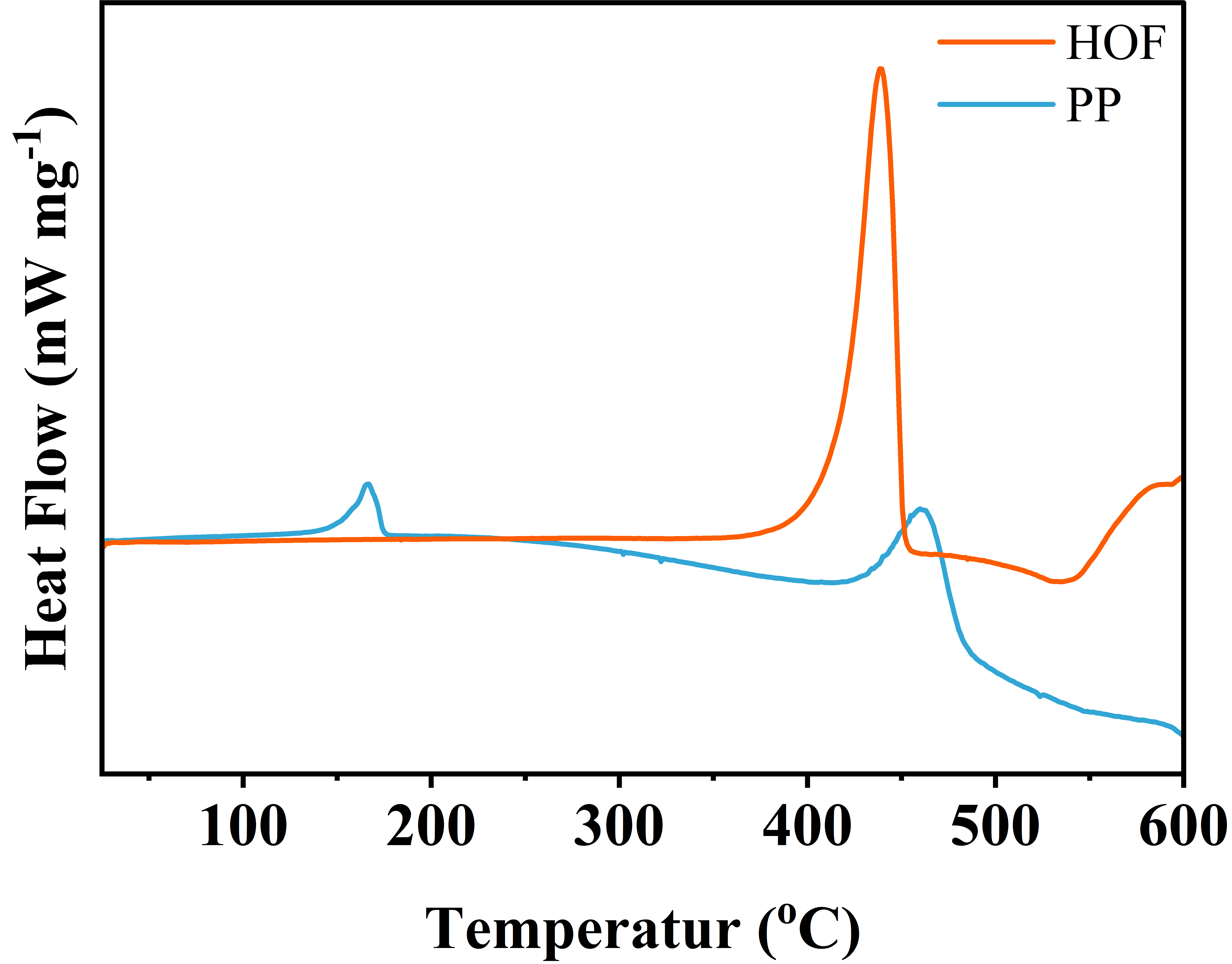


**Fig. S14** Differential scanning calorimetry of HOF and PP separator


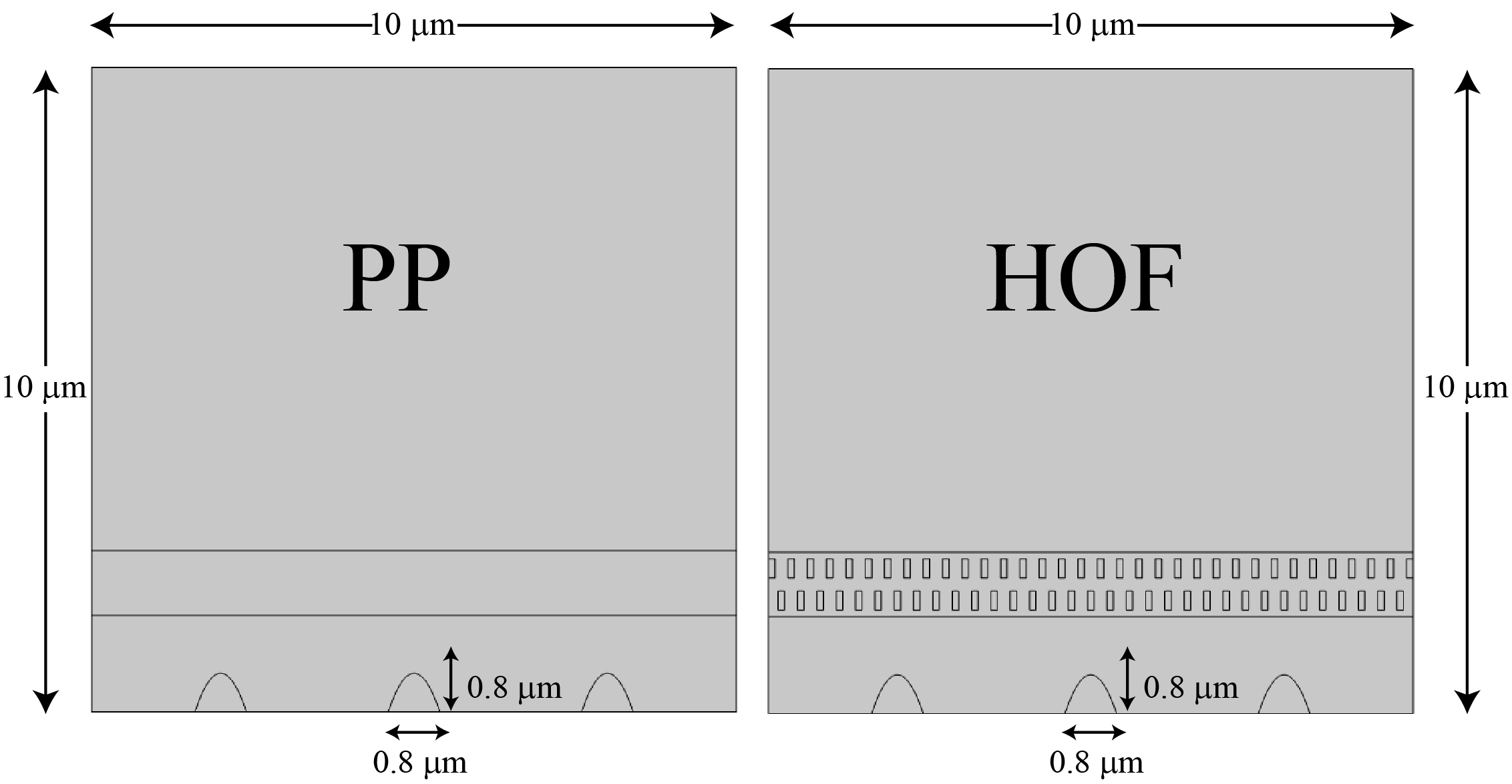


**Fig. S15** Model for COMSOL simulation


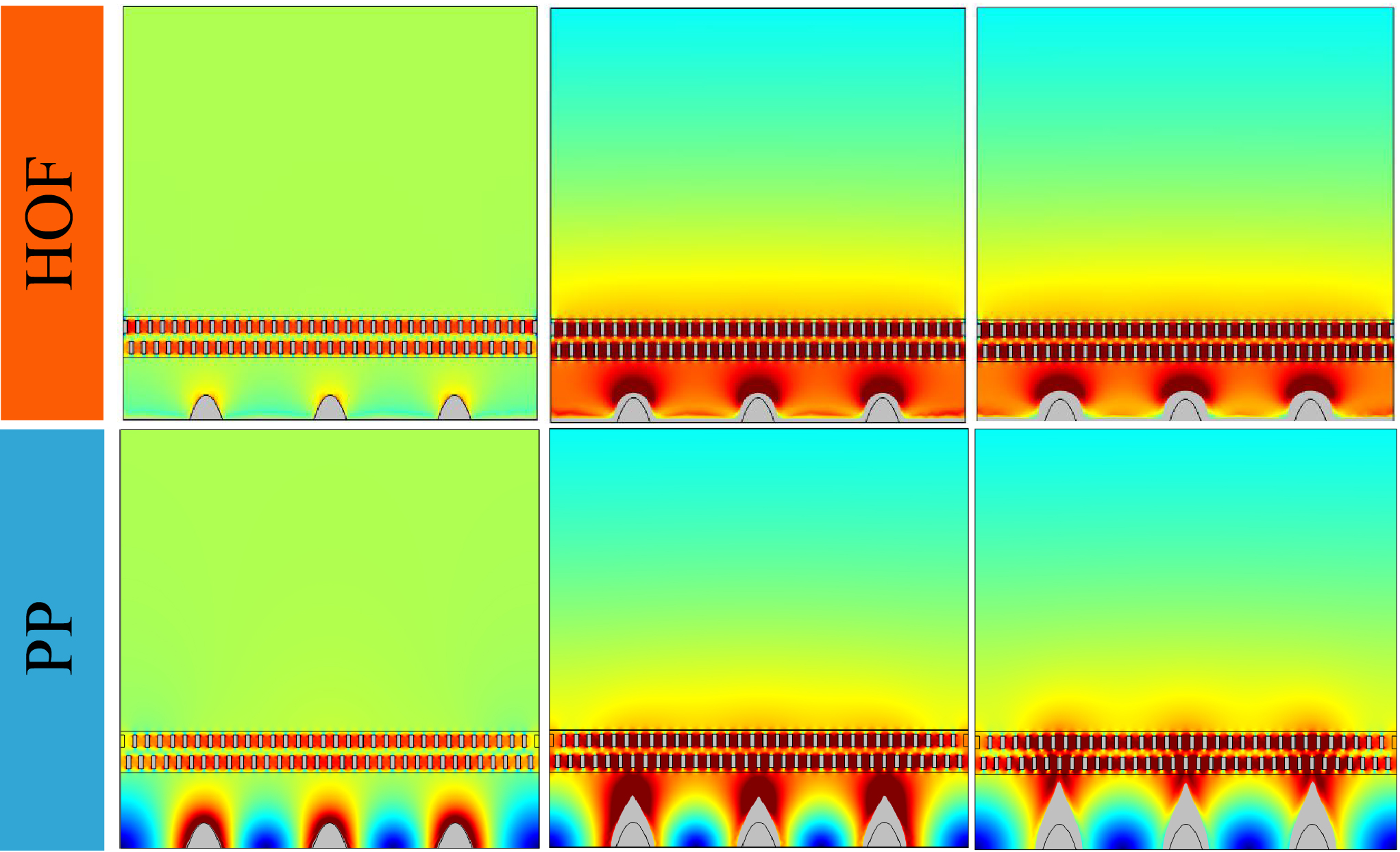


**Fig. S16** Na^+^ concentration field at the electrode interface in cells assembled with HOF and PP


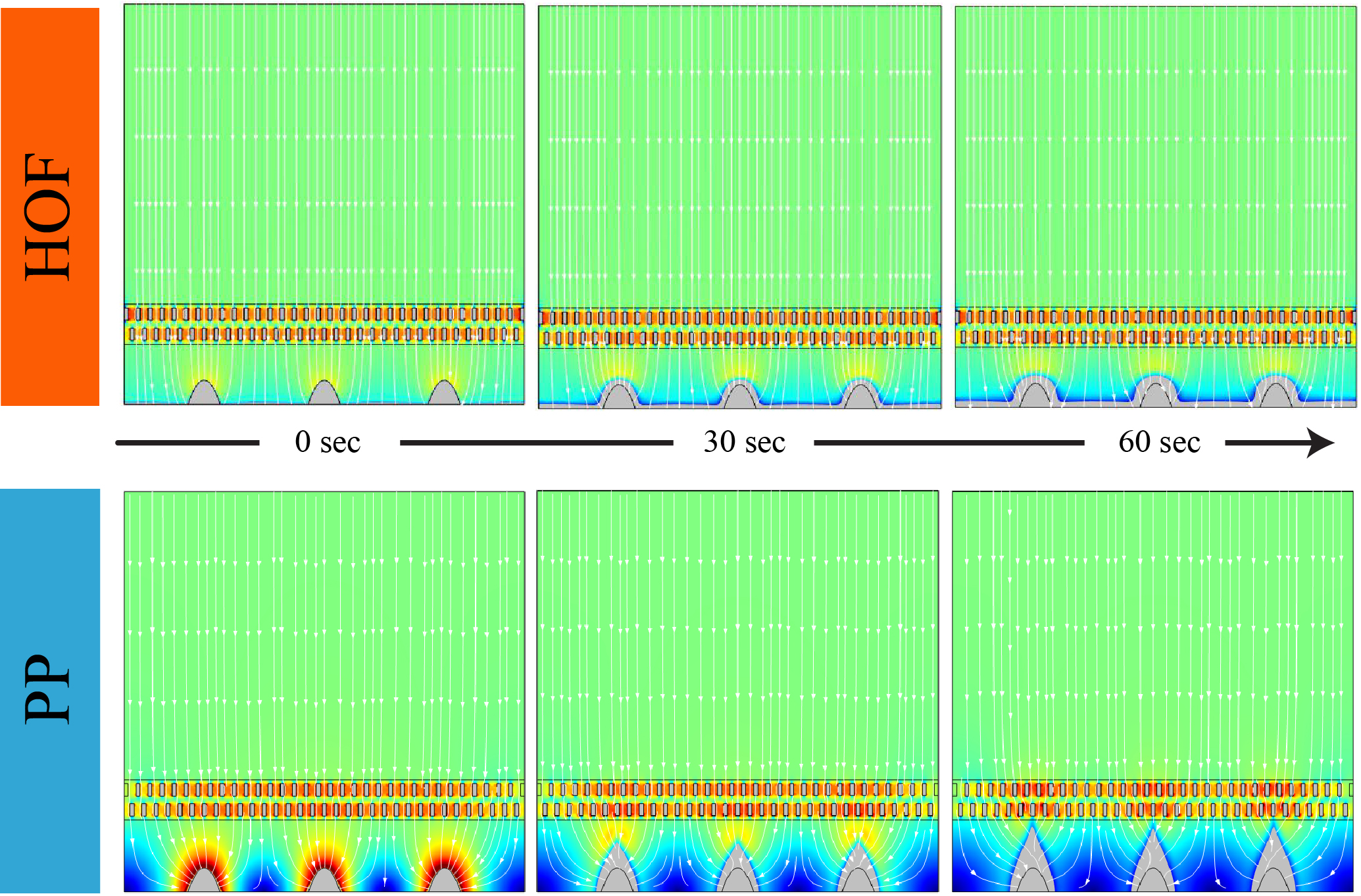


**Fig. S17** FEM simulation of the electrolyte current density distribution in a cell model with HOF and PP


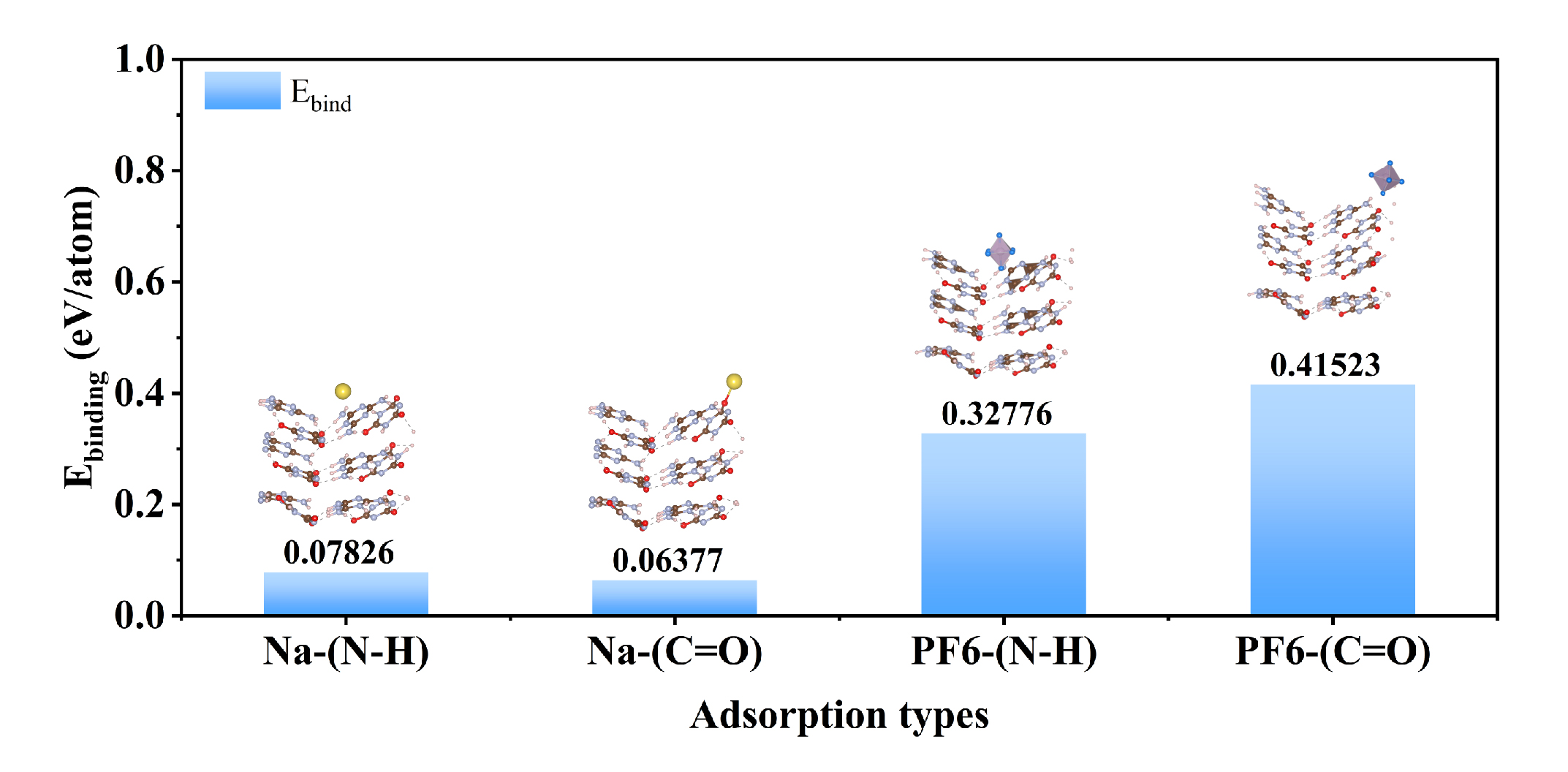


**Fig. S18** DFT-calculated binding energies between HOF functional groups (N–H, C=O) and ions (Na⁺, PF₆⁻)


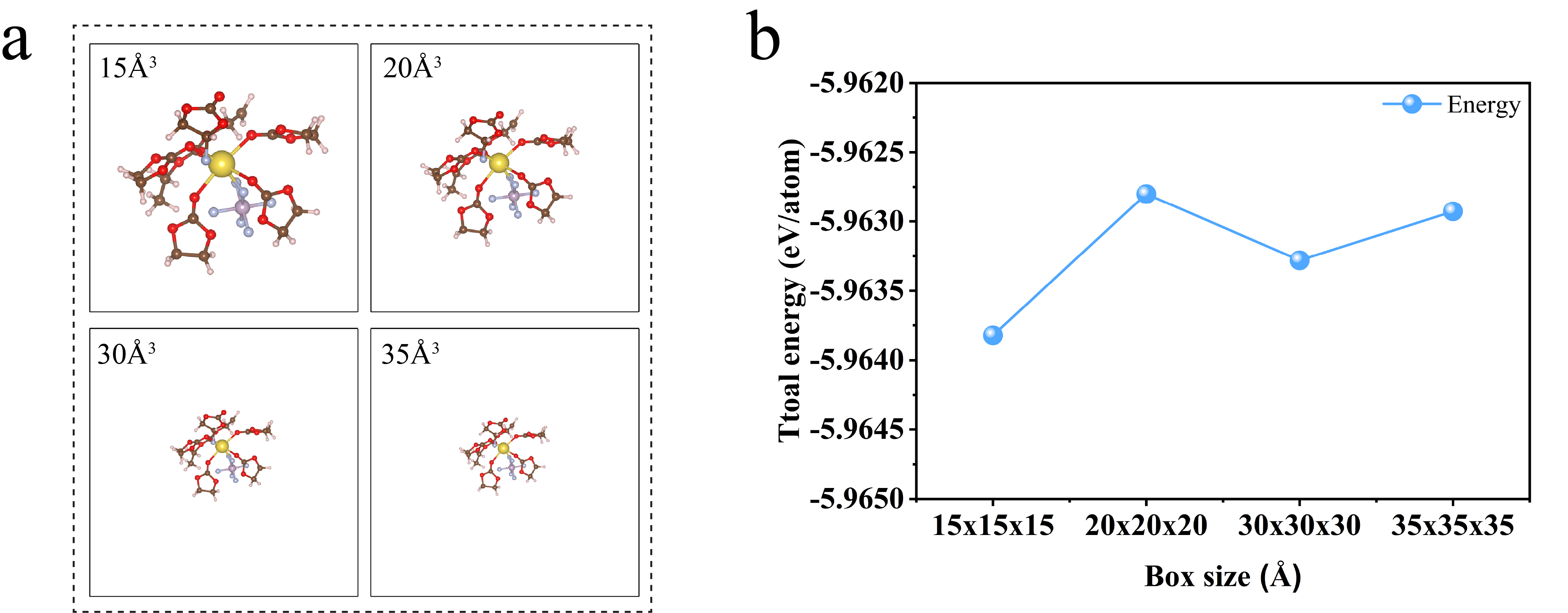


**Fig. S19** (a) optimized electrolyte configurations in cubic vacuum boxes of 15, 20, 30, and 35 Å; (b) total energy as a function of box size showing negligible energy variation at larger boxes (Γ-point).


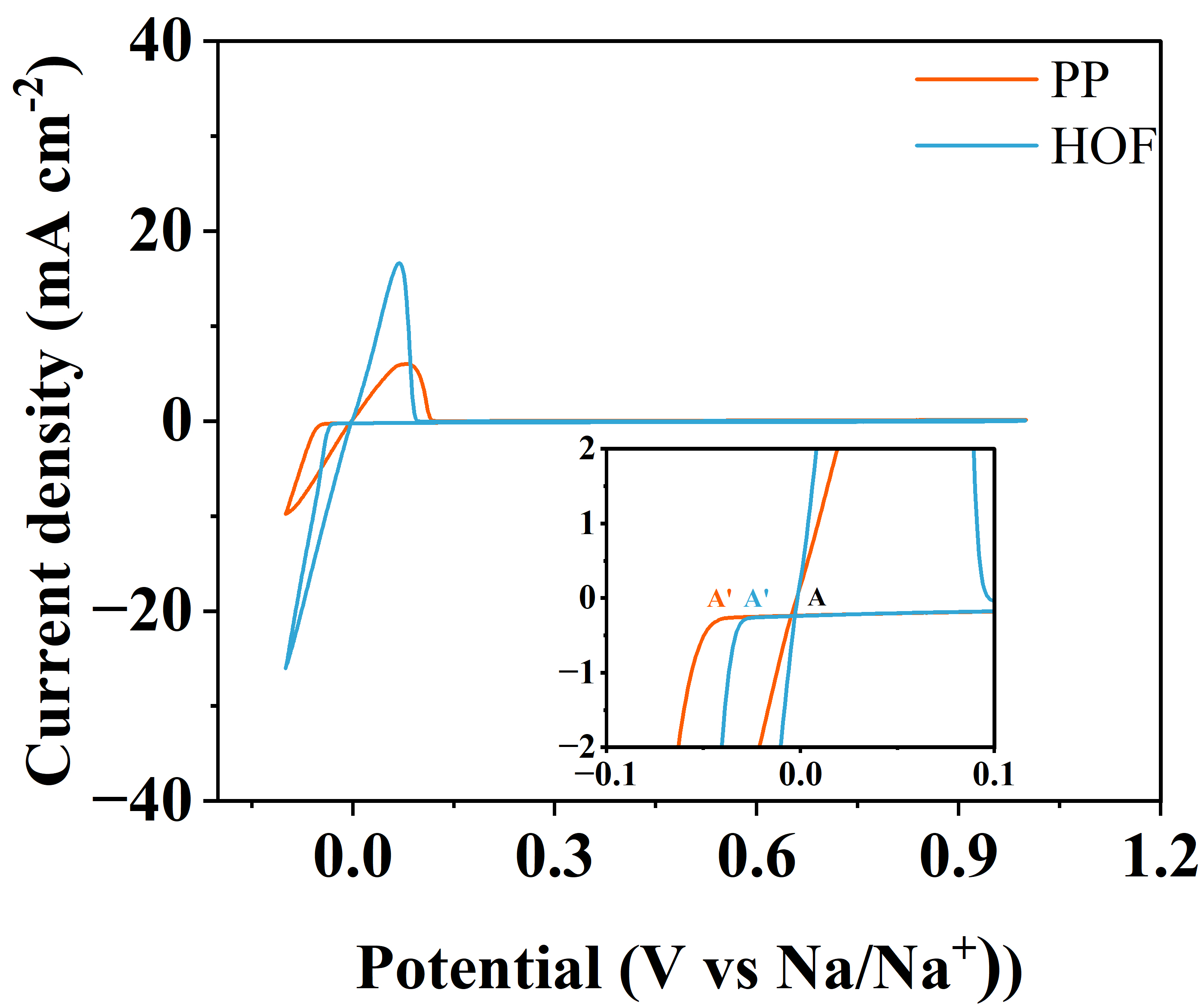


**Fig. S20** CV profiles of Na||Cu cell with different separators at 0.1mA s^-1^


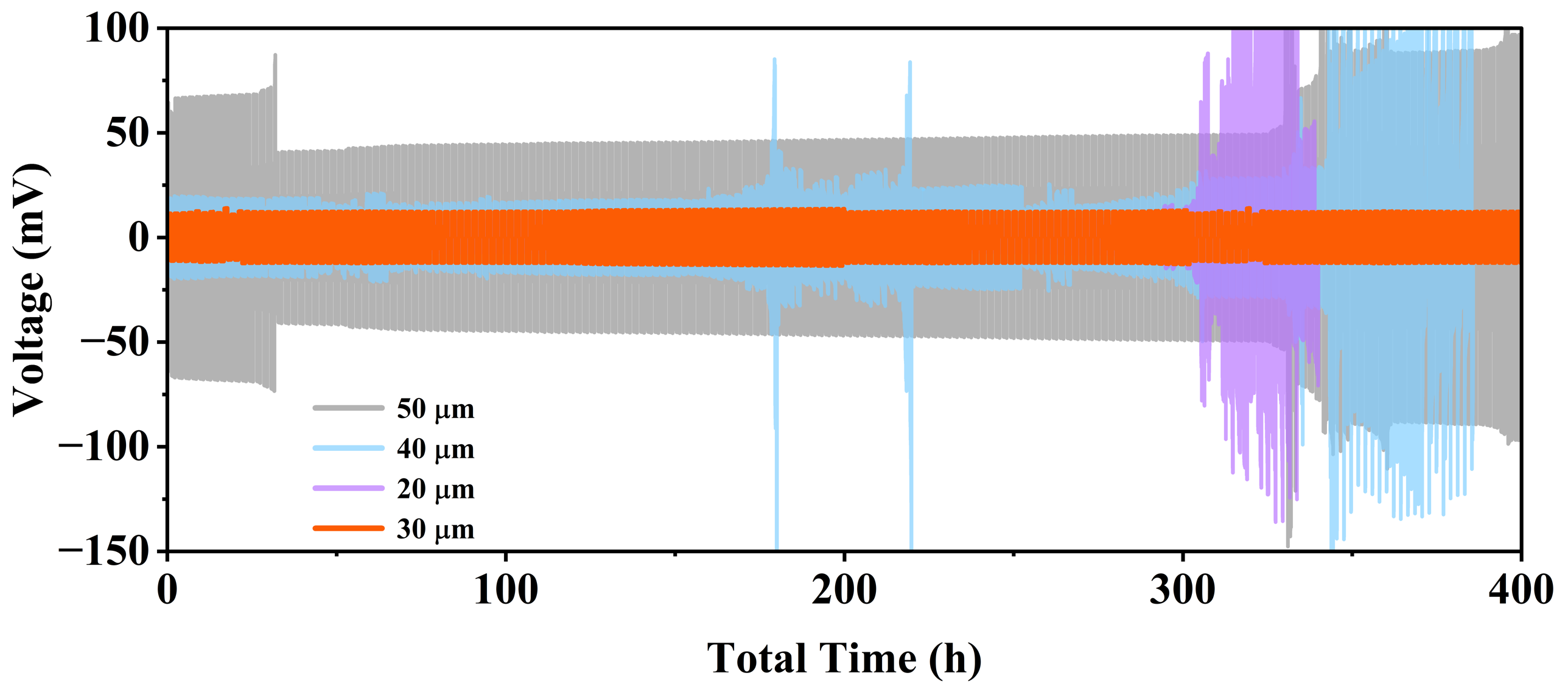


**Fig. S21** Long-term cycling of symmetric Na||Na cells at 60 °C at 1mA cm^-2^ and 1mAh cm^-2^ with different separator thickness


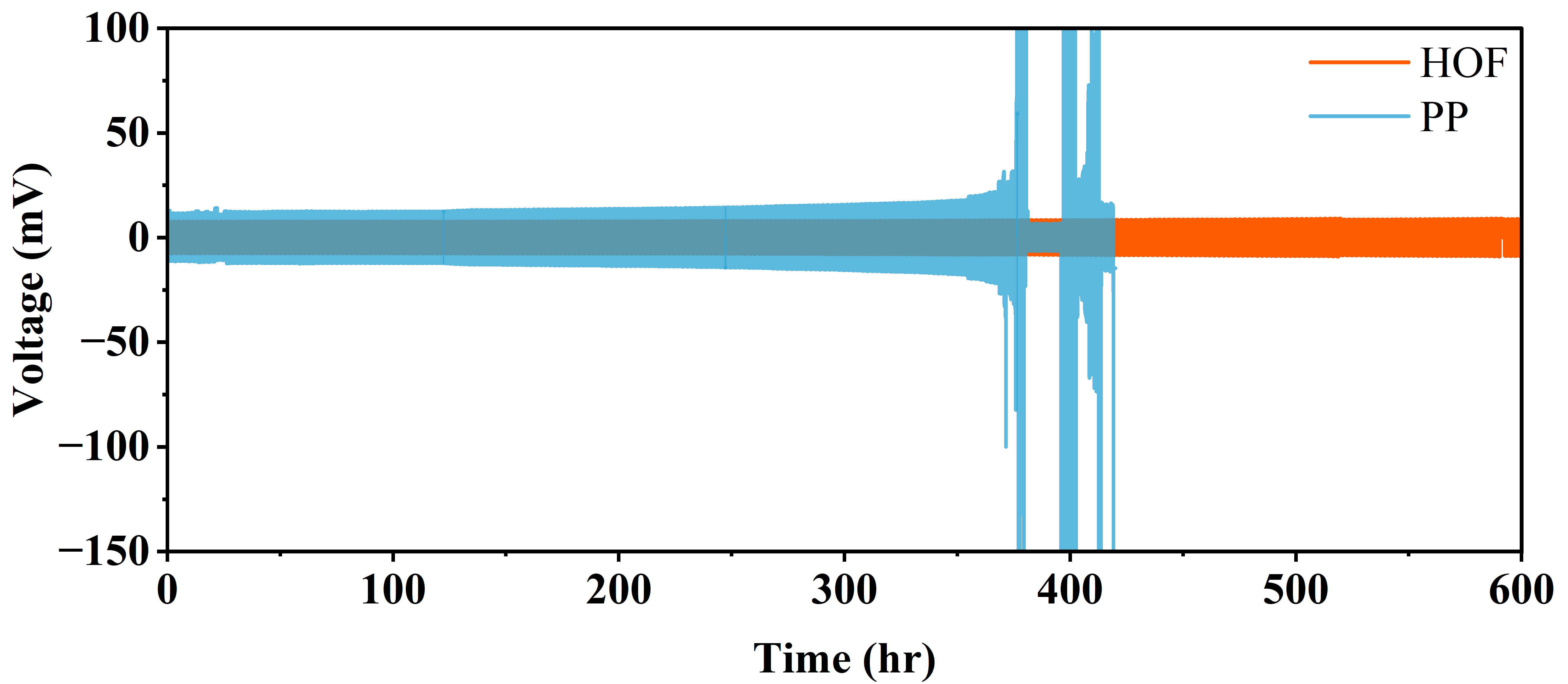


**Fig. S22** Long-term cycling of symmetric Na||Na cells at 25 °C at 1mA cm^-2^ and 1mAh cm^-2^


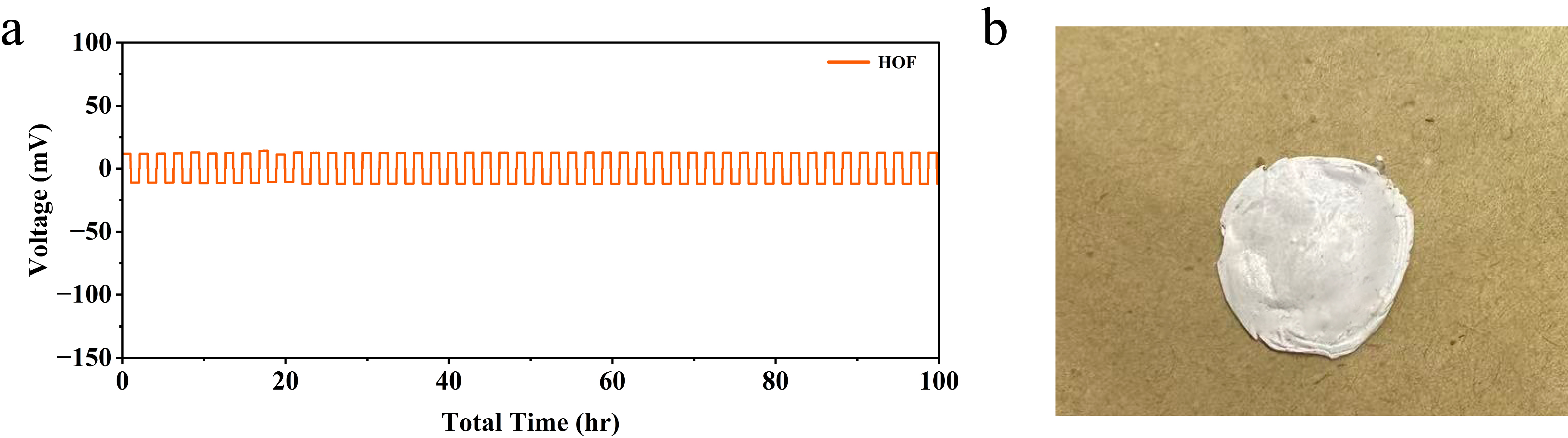


**Fig. S23** (a) Na||Na symmetrical cell performance with HOF separator at 1 mAh cm^-2^ and 1 mAh cm^-2^ (b) Digital photo graph of cycled HOF separator


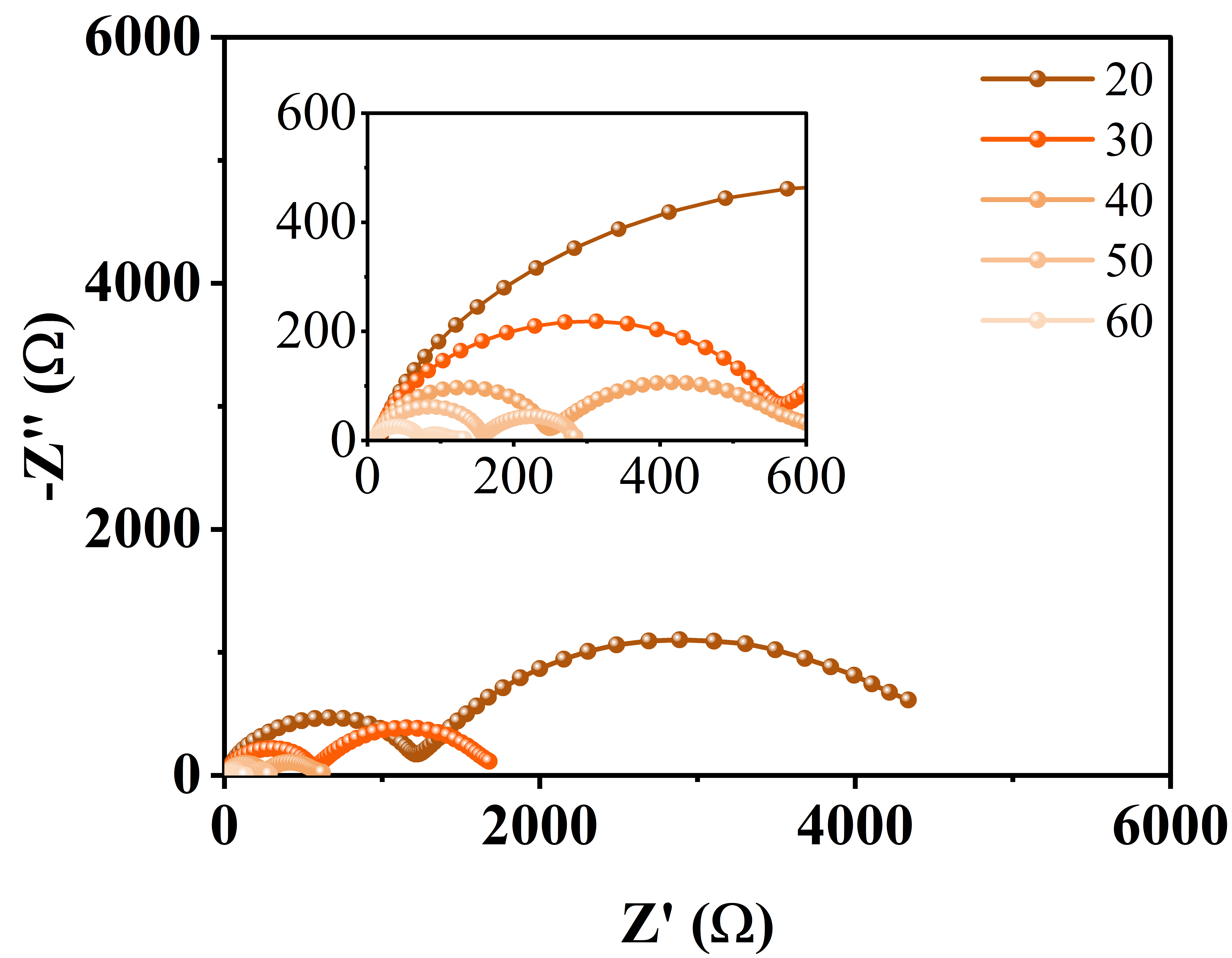


**Fig. S24** Nyquist plot of Na||Na cell with HOF at different temperatures


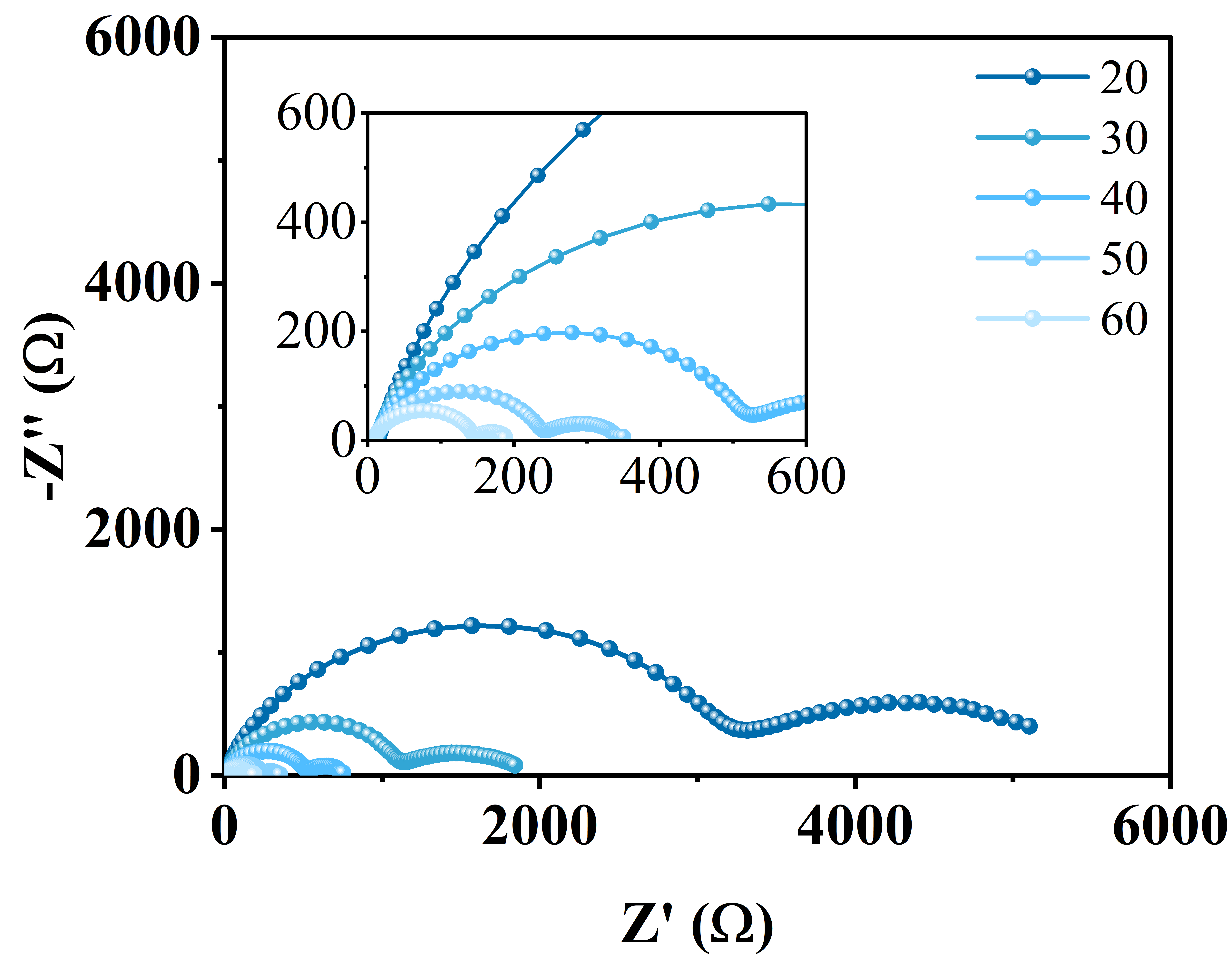


**Fig. S25** Nyquist plot of Na||Na cell with PP at different temperatures


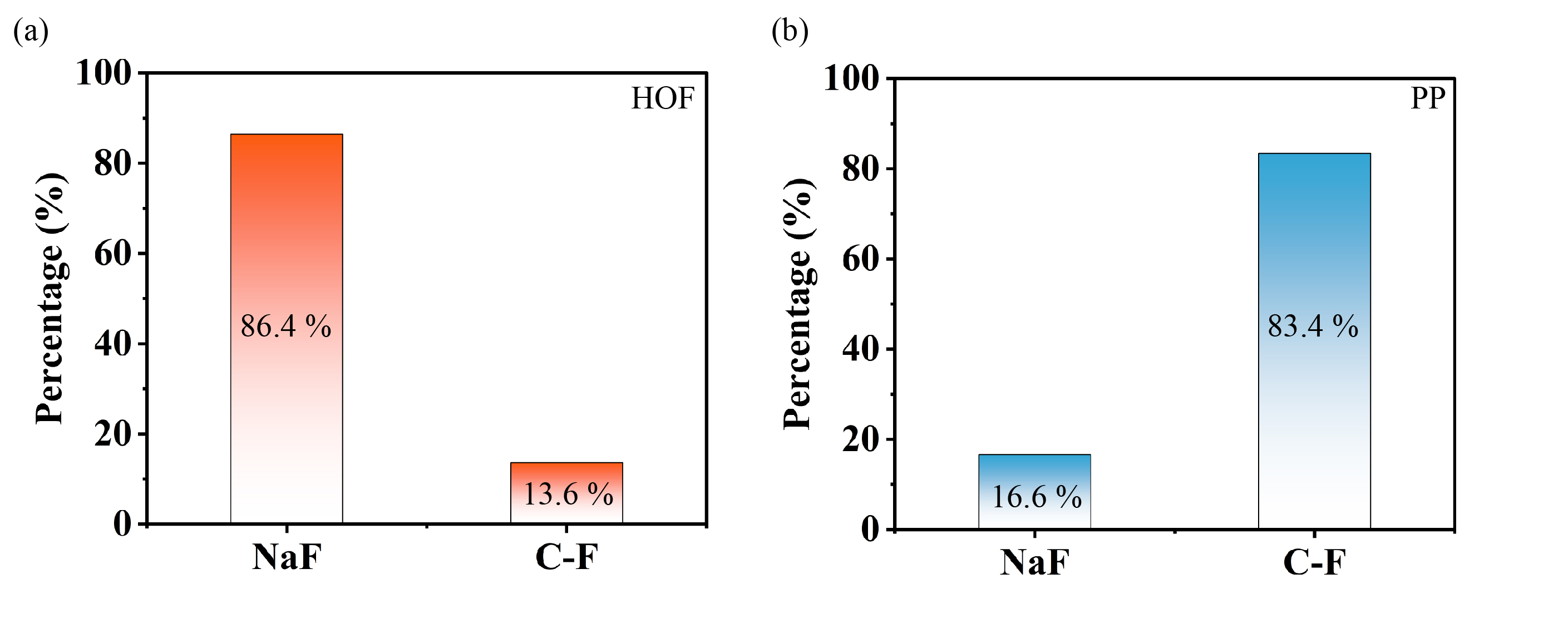


**Fig. S26** XPS spectra of SEI on metal anode cycled with PP separator


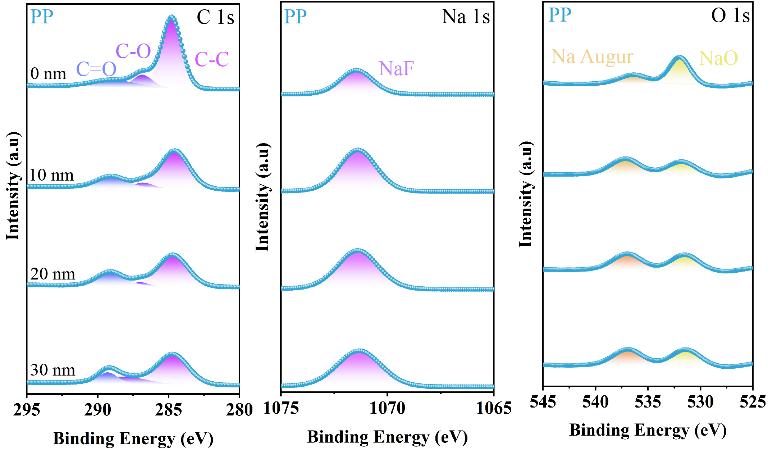


**Fig. S27** XPS spectra of SEI on metal anode cycled with PP separator


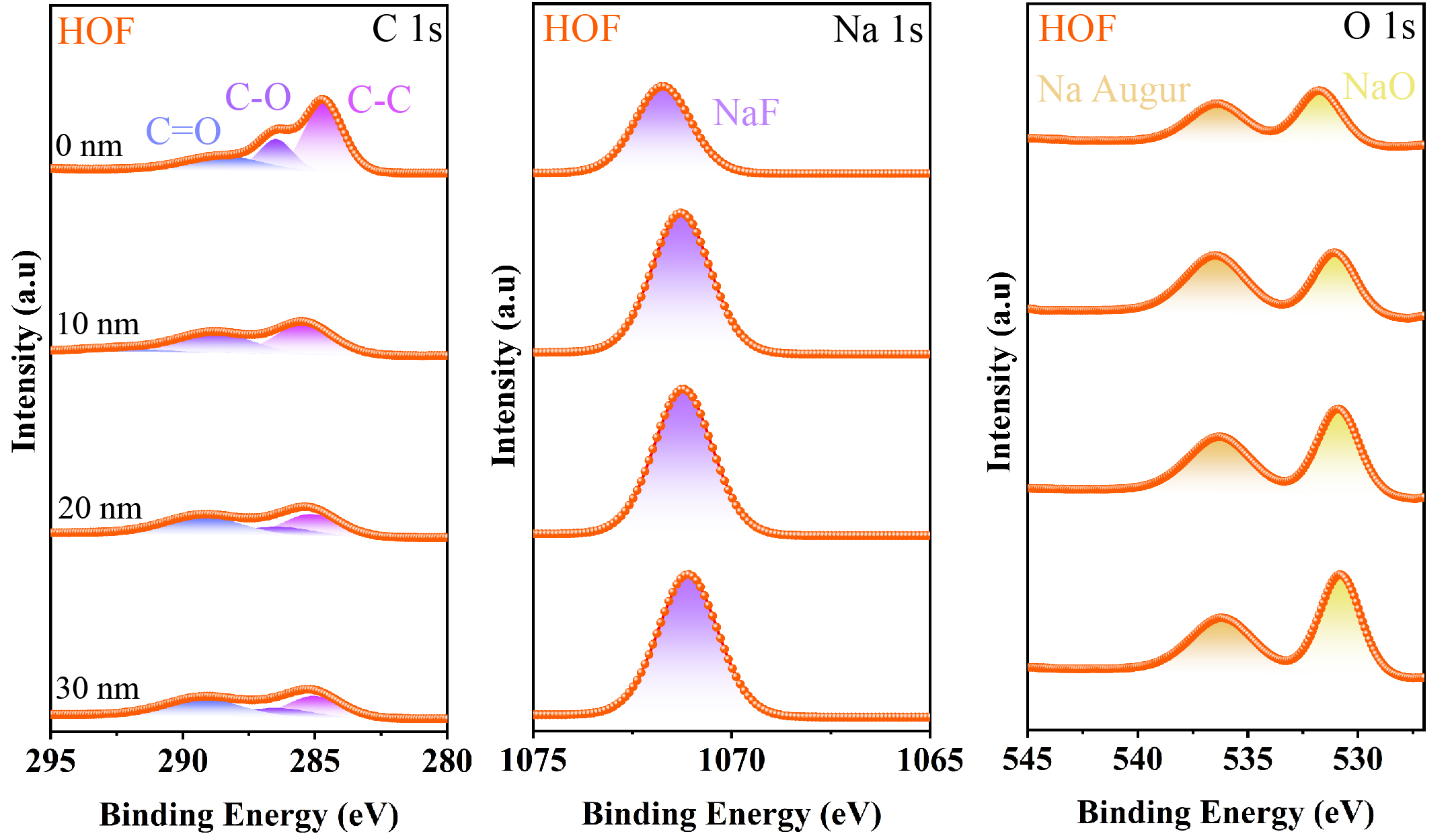


**Fig. S28** XPS spectra of SEI on metal anode cycled with HOF separator


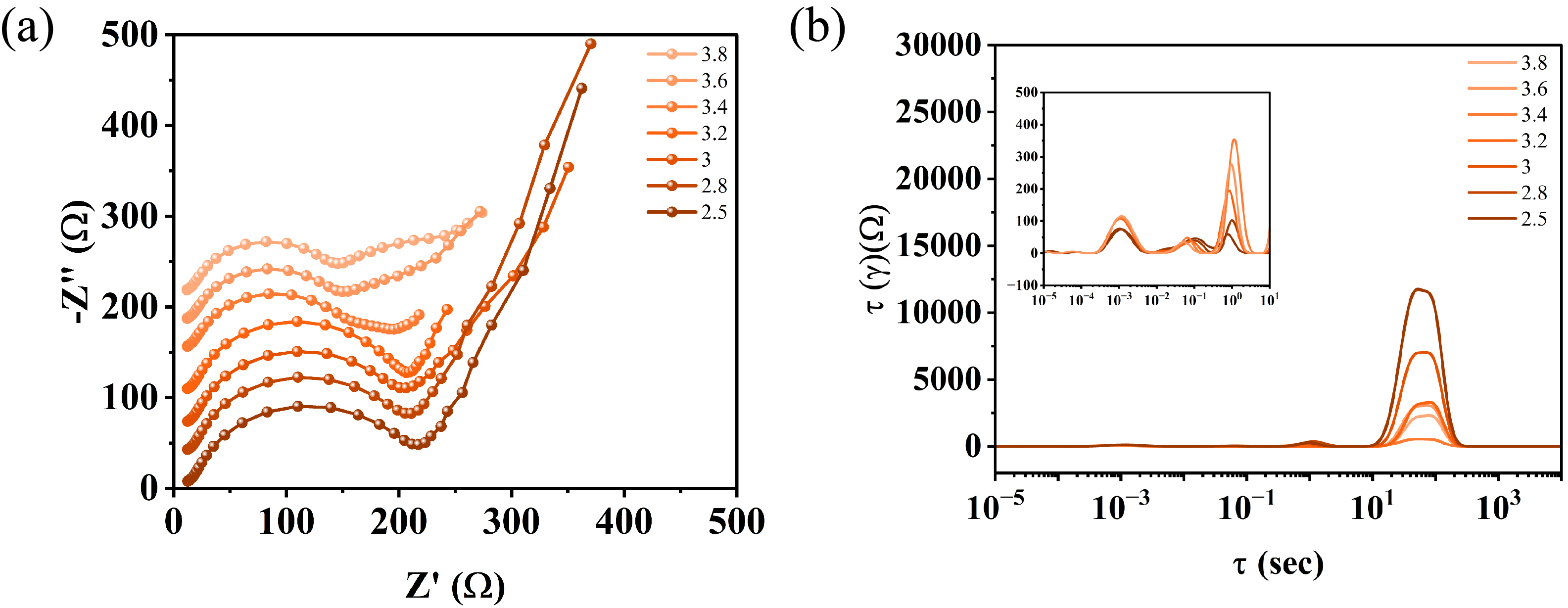


**Fig. S29 (a)** Nyquist plots obtained from IS-GEIS results of cell cycled (discharge) with HOF seprator at 0.1C with and eequidistance voltage 0.2V (**b**) corresponding DRT trasnformation of IS-GEIS

*
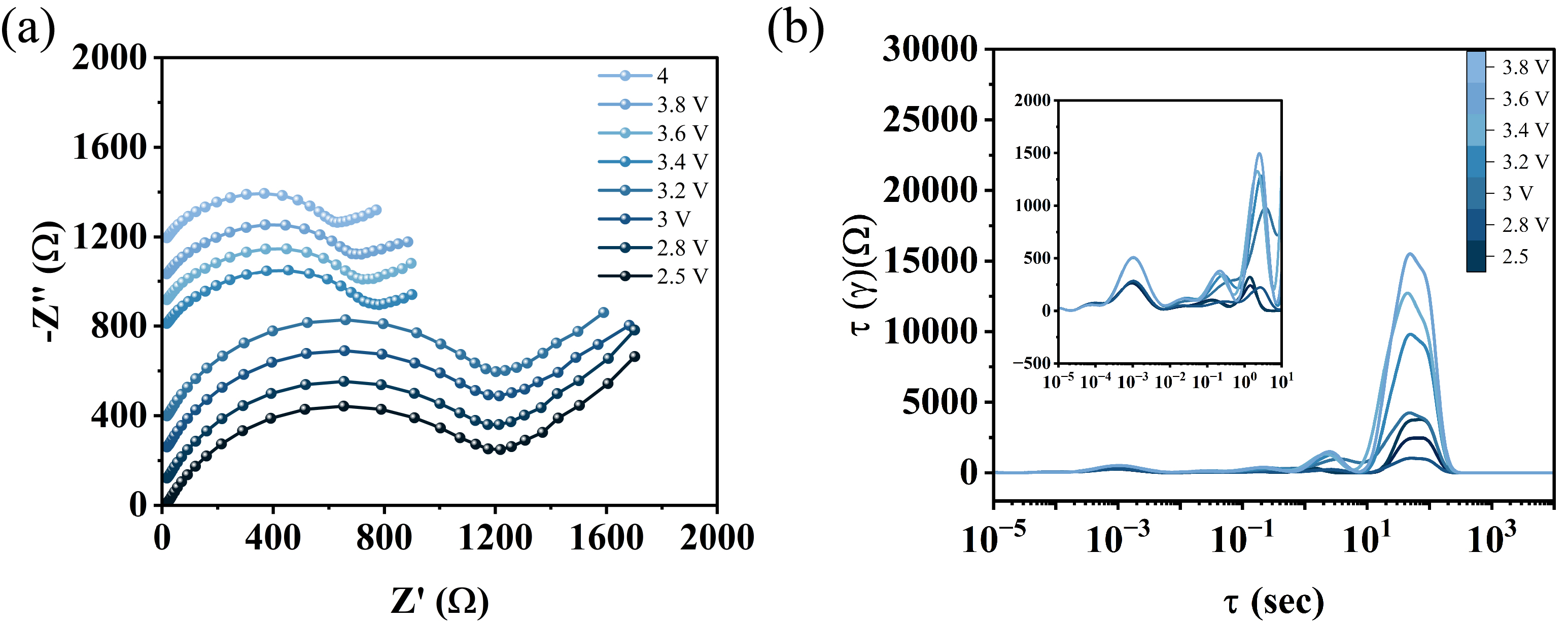
*

**Fig. S30 (a)** Nyquist plots obtained from IS-GEIS results of cell cycled (discharge) with PP seprator at 0.1C with and eequidistance voltage 0.2V (**b**) corresponding DRT trasnformation of IS-GEIS


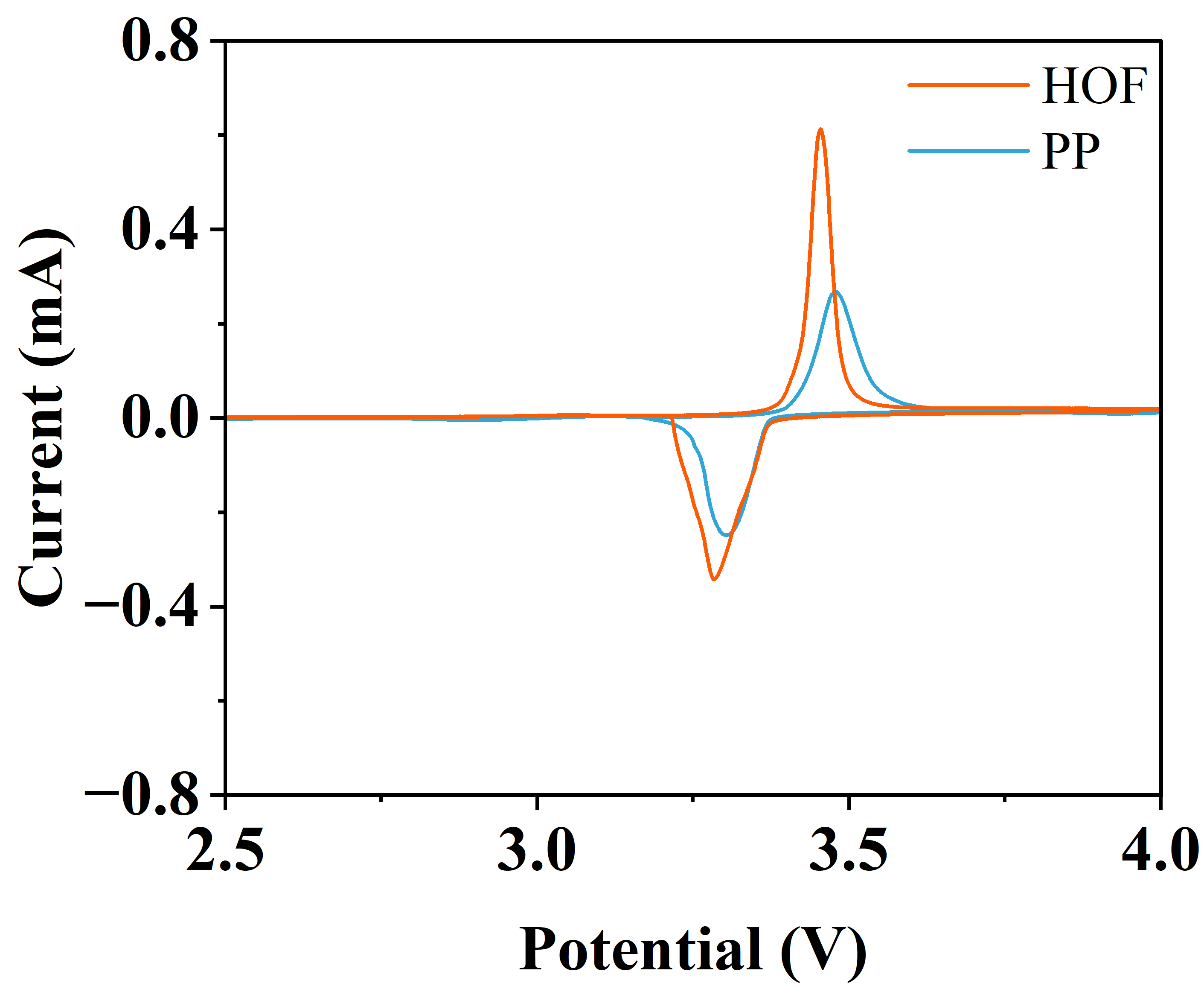


**Fig. S31** Cyclic Voltametery of Na||NVP with HOF and PP separators at 0.5 mV s^-1^


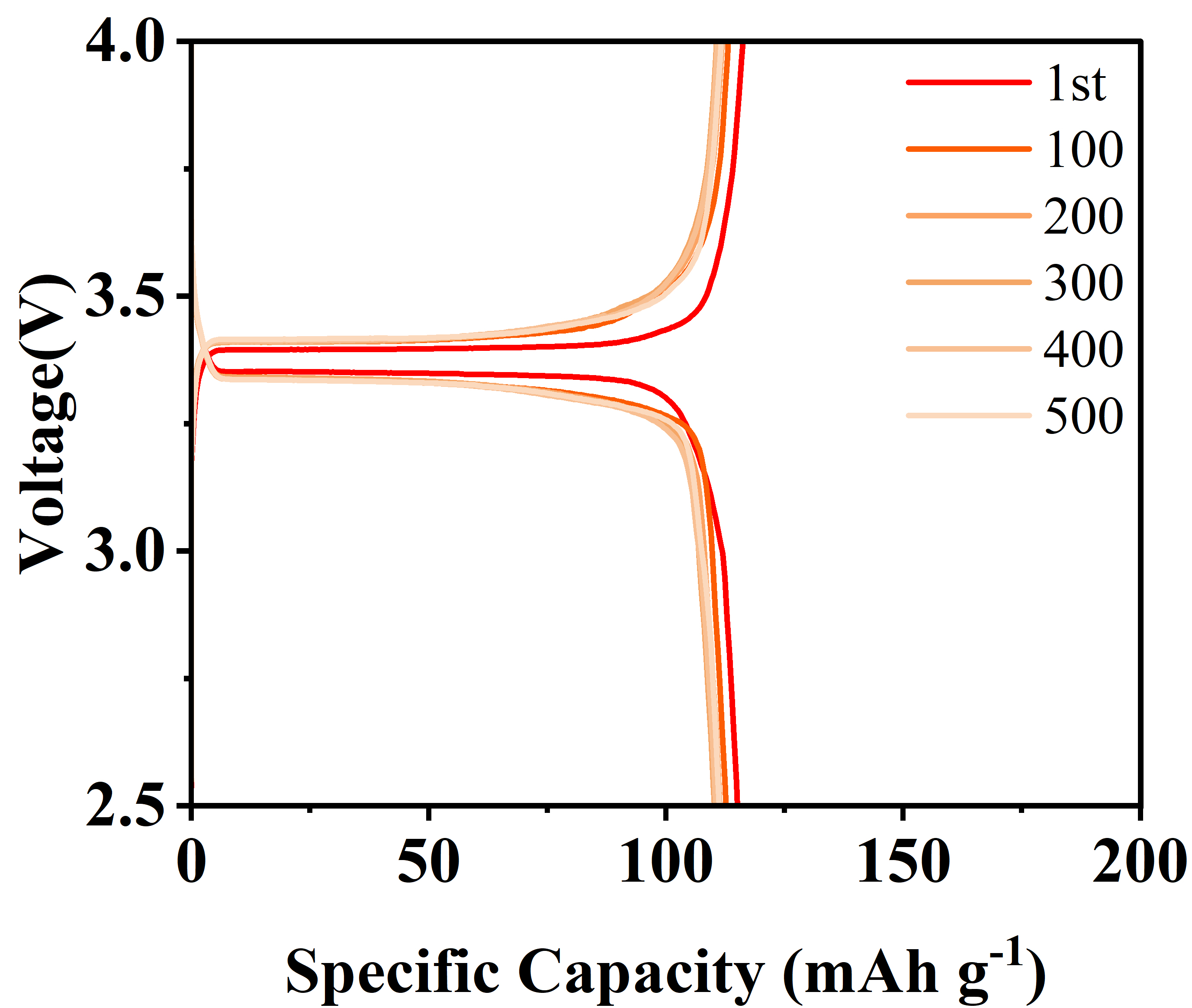


**Fig. S32** Charge-discharge curve of Na||NVP with HOF seprator cycled at 60^o^C at 5C (1C = 120 mAh g^-1^)


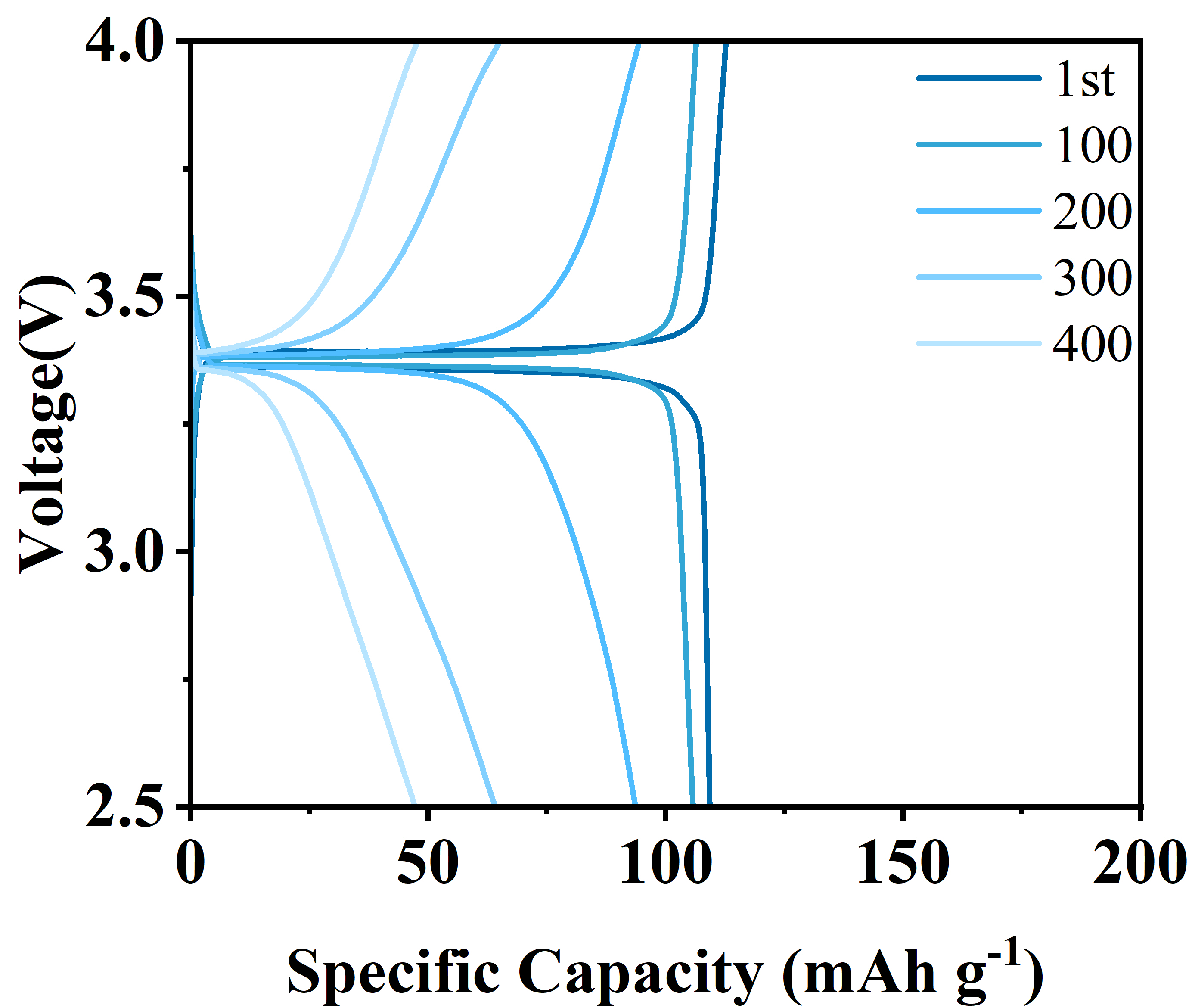


**Fig. S33** Charge-discharge curve of Na||NVP with PP seprator cycled at 60^o^C at 5C (1C = 120 mAh g^-1^)


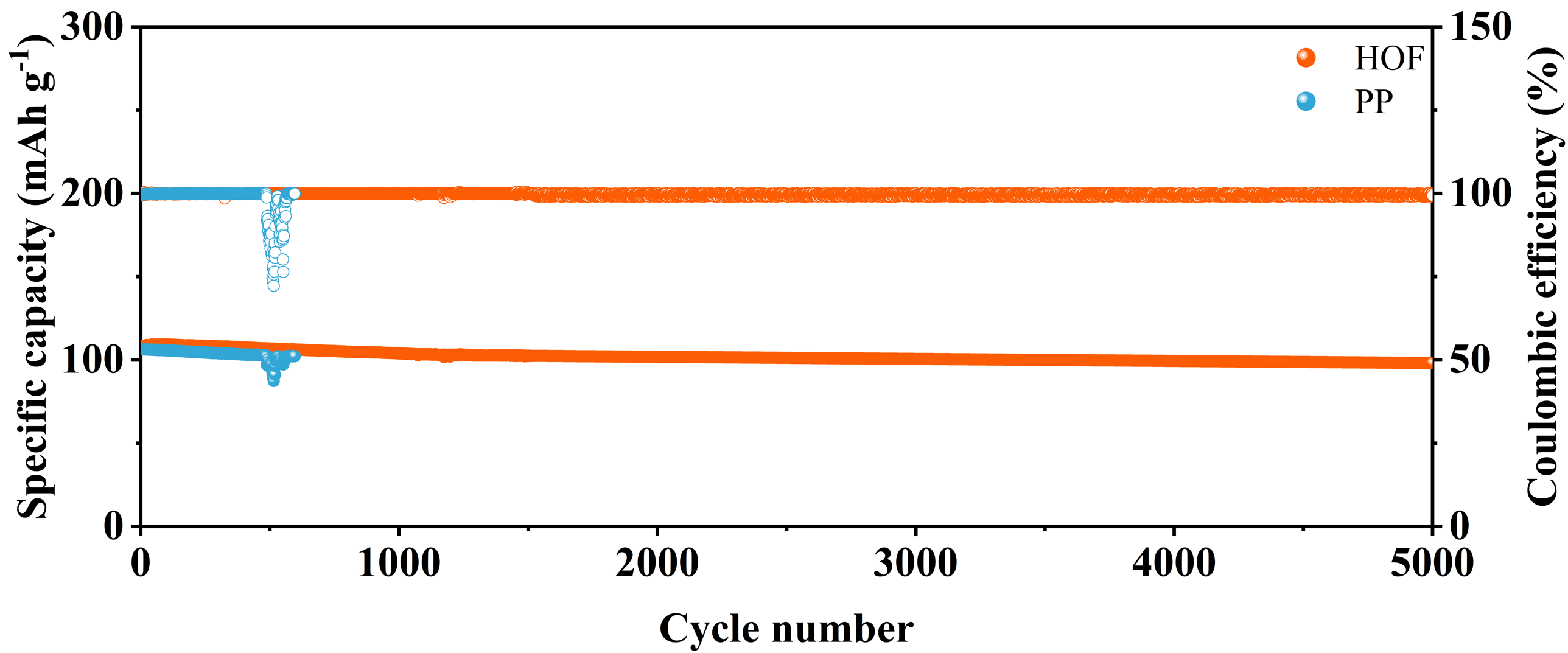


**Fig. S34** Long-term cycling stability at 25 °C and 5 C


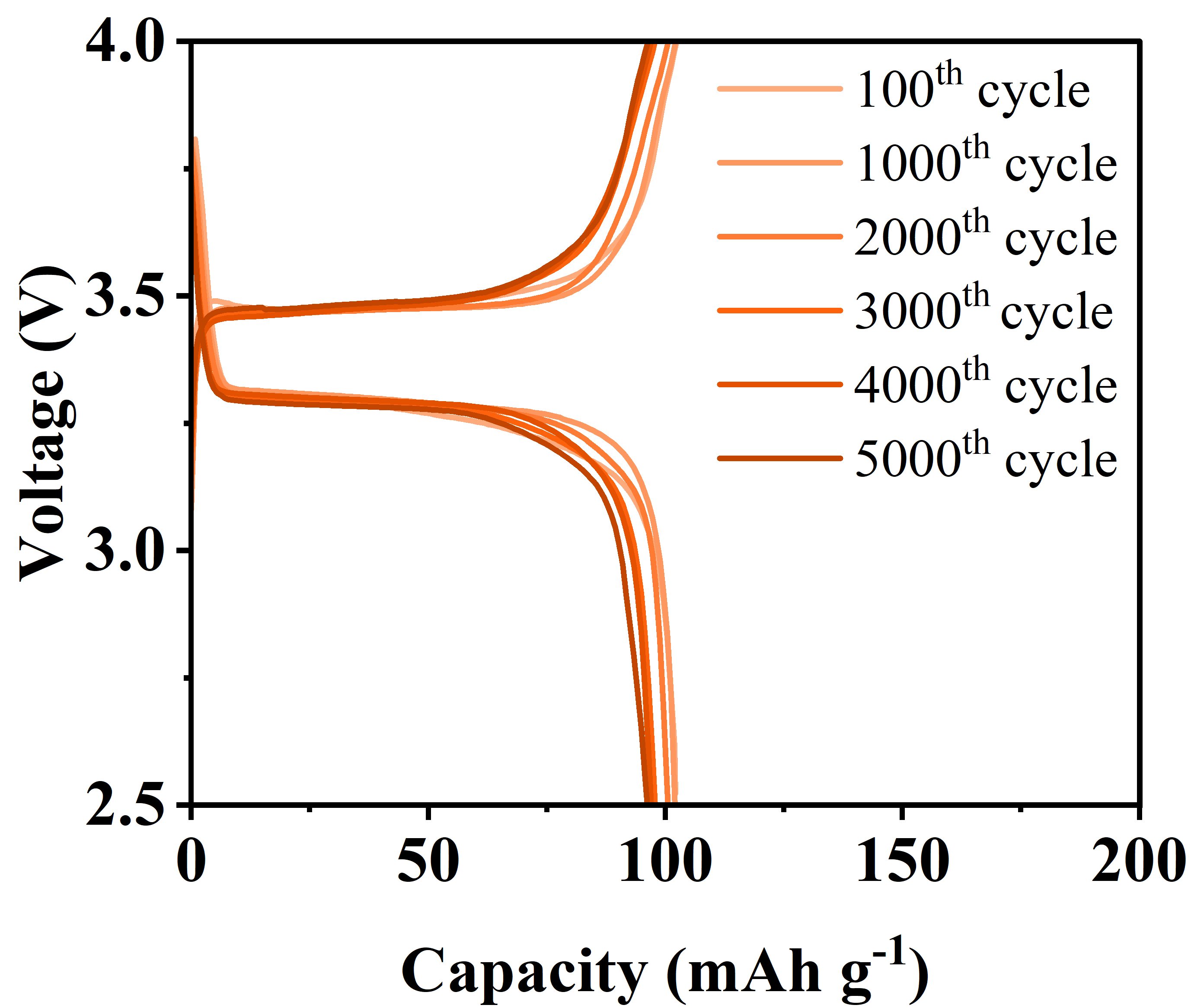


**Fig. S35** Charge-discharge curve of Na||NVP with HOF seprator cycled at 25 ^o^C at 5C (1C = 120 mAh g^-1^)


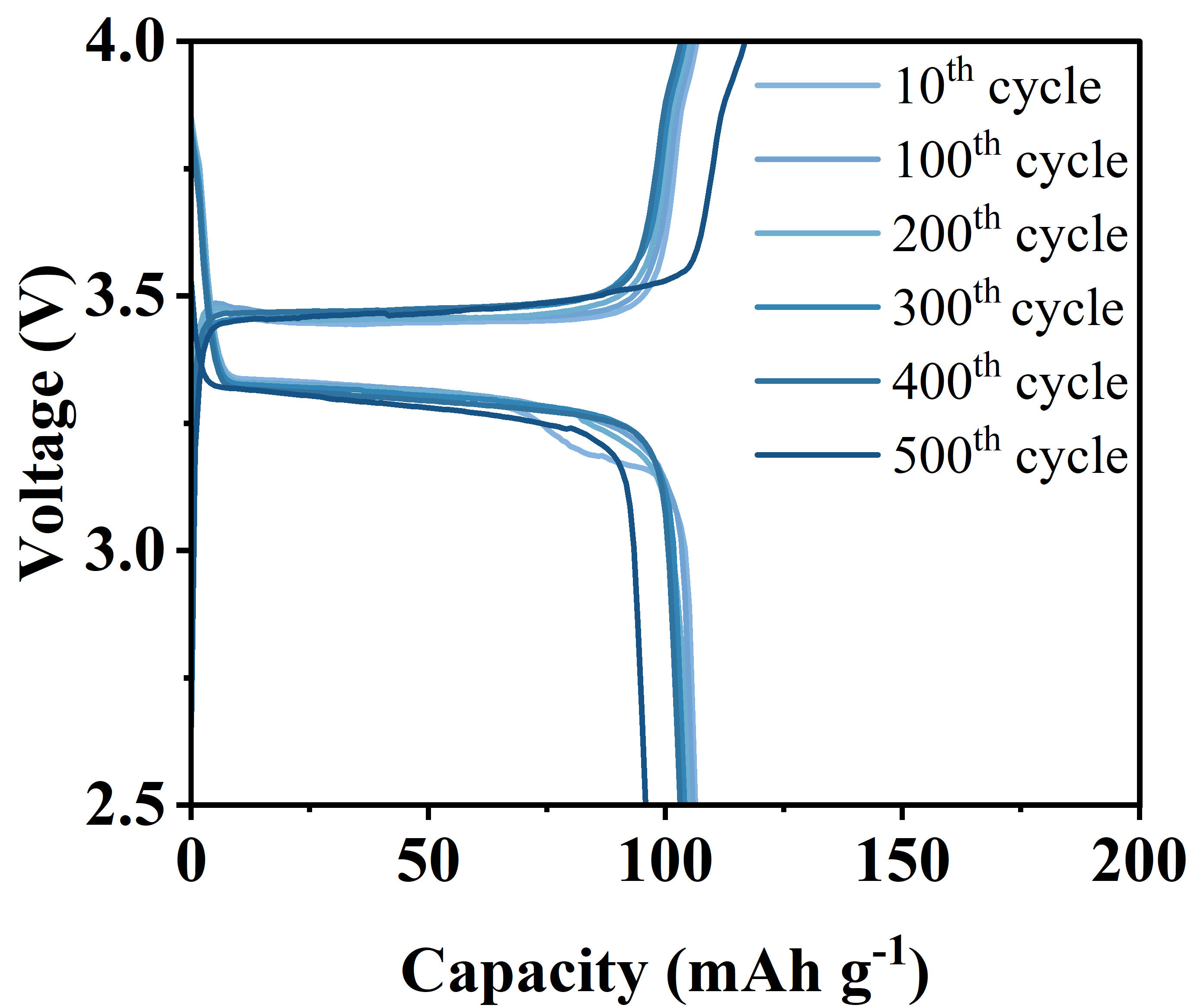


**Fig. S36** Charge-discharge curve of Na||NVP with HOF seprator cycled at 25 ^o^C at 5C (1C = 120 mAh g^-1^)

**Table S1**: Comparison of different parameters for reported separators

| **Separator** | **Ionic Conductivity (mS cm^-1^)** | **t_Na_^+^** | **Na ^+^ Contribution** | **Refs** |
| --- | --- | --- | --- | --- |
| HOF | 1.47 | 0.92 | 1.35 | This work |
| COF@PP | 1.13 | 0.82 | 0.93 | [S10] |
| Co-NWS | 1.39 | 0.83 | 1.16 | [S11] |
| Zr-O complex coating layer | 1.05 | 0.78 | 0.82 | [S12] |
| Bis[S(3-methyldimethoxysilyl)propyl]-  polypropylene oxide (BSPPO) coating on commercial substrates | 1.33 | 0.69 | 0.92 | [S13] |

**Table S2** Comparison of Na∥Na symmetric-cell performance with prior separator reports

| Strategies | Current density (mA cm^-2^ ) | Areal capacity (mAh cm^-2^ ) | Cycle life (h) | Electrolytes | Refs |
| --- | --- | --- | --- | --- | --- |
| OHTAPQ@Na | 2 | 2 | 1500 | 1 M NaPF_6_/DEGDME | [S14] |
| Co-NWS | 0.5 | 0.5 | 500 | 1.0 m NaPF_6_ in PC:EC = 1:1 v/v, with 10 wt% FEC | [S11] |
| UFS2@GF | 1 | 2 | 500 | 1.0 M NaFSI in PC:EC = 1:1 v/v, with 5 wt% FEC | [S15] |
| Nylon 6-cellulose acetate | 0.5 | 0.5 | 1000 | 1 m NaPF_6_ EC/DEC wt 5 wt.% FEC | [S16] |
| S-3500 separators | 1 | 0.5 | 1000 | 1 M NaClO4 in EC/DMC | [S17] |
| HOF separator | 2 | 2 | 2000 | 1 m NaPF_6_ EC/DEC wt 10 wt.% FEC | This work |

**Table S3** model parameter settings and boundary conditions

| C_init_ | 1 M | Initial concentration |
| --- | --- | --- |
| T_0_ | 298 K | System temperature |
| I | 2.5 mA cm^-2^ | Average current density |
| phis_cathode | -0.1 V | Cathode potential |
| alpha_c | 0.5 [S1] | Symmetry factor |
| alpha_a | 0.5 [S1] | Symmetry factor |
| MNa | 22.9898 g mol^-1^ | Molar mass of Na |
| rhoNa | 0.968 g cm^-3^ | Density of Na |
| D_Na_pp | 2.19e-12 cm^2^s^-1^ | Diffusivity |
| D_Na_HOF | 4.08e-12 cm^2^s^-1^ | Diffusivity |
| k_Na_pp | 1.08 mS cm^-1^ | Ionic conductivity |
| k_Na_HOF | 1.57 mS cm^-1^ | Ionic conductivity |

# Supplementary References

1. A.P. Thompson, H.M. Aktulga, R. Berger, D.S. Bolintineanu, W.M. Brown et al., LAMMPS - a flexible simulation tool for particle-based materials modeling at the atomic, meso, and continuum scales. Comput. Phys. Commun. **271**, 108171 (2022). <https://doi.org/10.1016/j.cpc.2021.108171>
2. G.A. Kaminski, R.A. Friesner, J. Tirado-Rives, W.L. Jorgensen, Evaluation and reparametrization of the OPLS-AA force field for proteins *via* comparison with accurate quantum chemical calculations on peptides. J. Phys. Chem. B **105**(28), 6474–6487 (2001). <https://doi.org/10.1021/jp003919d>
3. J. Hafner, Ab-initio simulations of materials using VASP: Density-functional theory and beyond. J. Comput. Chem. **29**(13), 2044–2078 (2008). <https://doi.org/10.1002/jcc.21057>
4. P.E. Blöchl, Projector augmented-wave method. Phys. Rev. B **50**(24), 17953–17979 (1994). <https://doi.org/10.1103/physrevb.50.17953>
5. J.P. Perdew, K. Burke, M. Ernzerhof, Generalized gradient approximation made simple. Phys. Rev. Lett. **77**(18), 3865–3868 (1996). <https://doi.org/10.1103/physrevlett.77.3865>
6. S. Grimme, Semiempirical GGA-type density functional constructed with a long-range dispersion correction. J. Comput. Chem. **27**(15), 1787–1799 (2006). <https://doi.org/10.1002/jcc.20495>
7. H.J. Monkhorst, J.D. Pack, Special points for Brillouin-zone integrations. Phys. Rev. B **13**(12), 5188–5192 (1976). <https://doi.org/10.1103/physrevb.13.5188>
8. S. Grimme, J. Antony, S. Ehrlich, H. Krieg, A consistent and accurate *ab initio* parametrization of density functional dispersion correction (DFT-D) for the 94 elements H-Pu. J. Chem. Phys. **132**(15), 154104 (2010). <https://doi.org/10.1063/1.3382344>
9. G. Henkelman, B.P. Uberuaga, H. Jónsson, A climbing image nudged elastic band method for finding saddle points and minimum energy paths. J. Chem. Phys. **113**(22), 9901–9904 (2000). <https://doi.org/10.1063/1.1329672>
10. M. Ali, H. Hussain, M. Ali, S. Aman, W. Yang et al., Regulating a NaF-rich SEI layer for dendrite-free sodium metal batteries using trifunctional halogenated covalent organic framework separators. Adv. Sci. **12**(37), e03693 (2025). <https://doi.org/10.1002/advs.202503693>
11. H. Liu, X. Zheng, Y. Du, M.C. Borrás, K. Wu et al., Multifunctional separator enables high-performance sodium metal batteries in carbonate-based electrolytes. Adv. Mater. **36**(5), e2307645 (2024). <https://doi.org/10.1002/adma.202307645>
12. Y. Zhang, S. Yang, S. Liu, Z. Yuan, J. Deng et al., Enabling anion-anchoring and anti-degradation cellulose separators for sodium metal batteries. Energy Storage Mater. **81**, 104463 (2025). <https://doi.org/10.1016/j.ensm.2025.104463>
13. Z. Hou, W. Zhang, X. Kong, Q. Ren, Z. Wang et al., Polypropylene oxide-modified cellulose separators for high-rate Na+ transport *via* a dual-pathway mechanism in sodium-ion batteries. Chem. Eng. J. **513**, 162839 (2025). <https://doi.org/10.1016/j.cej.2025.162839>
14. Z. Shen, Z. Bo, R. Shi, L. Liu, H. Li et al., Functional p-π conjugated organic layer empowers stable sodium metal batteries. Adv. Funct. Mater. **35**(19), 2420573 (2025). <https://doi.org/10.1002/adfm.202420573>
15. J. Lv, Z. Tang, Q. Zhang, H. Sun, M. OuYang et al., Synergistic dual-polar-functionalized metal–organic framework-modified separator for stable and high-performance sodium metal batteries. ACS Nano **19**(16), 16133–16146 (2025). <https://doi.org/10.1021/acsnano.5c04051>
16. S. Guo, W. Li, X. Wu, X. Guo, Z. Gong et al., Functional separator induced interface potential uniform reformation enabling dendrite-free metal batteries. Adv. Funct. Mater. **35**(37), 2504599 (2025). <https://doi.org/10.1002/adfm.202504599>
17. J. Wang, Z. Xu, Q. Zhang, X. Song, X. Lu et al., Stable sodium-metal batteries in carbonate electrolytes achieved by bifunctional, sustainable separators with tailored alignment. Adv. Mater. **34**(49), e2206367 (2022). <https://doi.org/10.1002/adma.202206367>
